# Comparison of 5-Day Multidaily Neuronavigated Theta-Burst Sessions With 6-Week Standard Repetitive Transcranial Magnetic Stimulation (the Dutch Depression Outcome Trial): Protocol for a Randomized Controlled Trial

**DOI:** 10.2196/70121

**Published:** 2025-08-21

**Authors:** Annemiek Dols, Tom Biemans, Coen Coomans, Philip van Eijndhoven, Iris Dalhuisen, Ysbrand D van der Werf, Chris Vriend, Elvira S Amaral Gomes, Alexander T Sack, Teresa Schuhmann, Mashood Chaudhry, Martijn Arns, Bethany Hipple Walters, Ben Wijnen, Andrew Zalesky, Robin Cash, Daniel M Blumberger, Karel WF Scheepstra, Adriaan W Hoogendoorn, Odile A van den Heuvel, Eric van Exel, The D-DOTT Consortium

**Affiliations:** 1 Department of Psychiatry Amsterdam UMC, Vrije Universiteit Amsterdam Amsterdam The Netherlands; 2 Department of Psychiatry University Medical Center Utrecht Utrecht The Netherlands; 3 Amsterdam Neuroscience Mood, Anxiety, Psychosis, Stress & Sleep Amsterdam The Netherlands; 4 Department of Psychiatry Radboud University Medical Center Nijmegen The Netherlands; 5 Donders Institute for Brain Cognition and Behavior, Centre for Medical Neuroscience Nijmegen The Netherlands; 6 Amsterdam Neuroscience Compulsivity, Impulsivity and Attention Amsterdam The Netherlands; 7 Department of Anatomy and Neurosciences Amsterdam UMC, Vrije universiteit Amsterdam Amsterdam The Netherlands; 8 Organisation for Depression Amersfoort The Netherlands; 9 Department of Cognitive Neuroscience Faculty of Psychology and Neuroscience Maastricht University Maastricht The Netherlands; 10 Department of Psychiatry, Maastricht University Medical Centre Maastricht The Netherlands; 11 Research Institute Brainclinics, Brainclinics Foundation Nijmegen The Netherlands; 12 Department of Psychiatry and Behavioral Sciences Stanford University Stanford, CA United States; 13 Trimbos Institute - Institute for Mental Health and Addiction Utrecht The Netherlands; 14 Melbourne Neuropsychiatry Centre and Department of Biomedical Engineering The University of Melbourne Melbourne Australia; 15 Temerty Centre for Therapeutic Brain Intervention Centre for Addiction and Mental Health Toronto, ON Canada; 16 Department of Psychiatry and Institute of Medical Science Faculty of Medicine University of Toronto Toronto, ON Canada; 17 Psychiatric Program of the Netherlands Brain Bank Netherlands Institute for Neuroscience Amsterdam The Netherlands; 18 Mental Health Program Amsterdam Public Health Amsterdam The Netherlands; 19 GGZ inGeest Amsterdam The Netherlands

**Keywords:** repetitive transcranial magnetic stimulation, rTMS, personalized accelerated intermittent theta-burst stimulation, personalized aiTBS, personalized functional connectivity–guided accelerated intermittent theta-burst stimulation, PAiT, Stanford neuromodulation therapy, SNT, functional magnetic resonance imaging, fMRI, dorsolateral prefrontal cortex, DLPFC, subgenual anterior cingulate cortex, sgACC, treatment-resistant depression

## Abstract

**Background:**

Novel therapies are crucial for patients with major depressive disorder, as more than 33% of patients do not respond to first-line treatments. A promising novel treatment strategy is an intensive 5-day course of personalized, functional connectivity–guided accelerated intermittent theta-burst stimulation (PAiT), modeled after the Stanford neuromodulation therapy protocol. This new form of repetitive transcranial magnetic stimulation (rTMS) may lead to higher remission rates in patients with treatment-resistant depression (TRD). However, it remains unclear how this accelerated strategy compares to the standard once-daily 10 Hz rTMS at inducing remission of depression.

**Objective:**

This study aims to compare cost-effectiveness using the PAiT protocol and the standard 10 Hz rTMS in patients with TRD.

**Methods:**

A total of 108 patients will be enrolled in this multicenter randomized controlled trial. Patients will receive stimulation over the left dorsolateral prefrontal cortex using either the PAiT protocol (10 sessions per day over 5 days, resulting in 50 sessions in total and 90,000 pulses) or standard 10 Hz rTMS (once daily for 6 weeks, resulting in 30 sessions in total and 90,000 pulses). Personalized targets will be identified in the accelerated intermittent theta-burst stimulation condition based on negative functional coupling between the subgenual anterior cingulate cortex and the dorsolateral prefrontal cortex. In the rTMS condition, the dorsolateral prefrontal cortex target locations are identified using the standard Beam-F3 method. In both conditions, coil placement is performed with neuronavigation, navigating either to the personalized functional connectivity target or the Beam-F3 location. Patients will undergo pre- and posttreatment functional magnetic resonance imaging scans, including cognitive and emotional tasks. Four follow-up assessments are scheduled at 7, 12, 26, and 31 weeks after baseline. We expect that the PAiT protocol is more cost-effective than the standard 10 Hz rTMS.

**Results:**

Recruitment for this randomized controlled trial started in February 2024. As of December 2024, we had enrolled 31 patients; the last participant is expected to complete their posttreatment assessments in January 2027.

**Conclusions:**

To our knowledge, this study is the first clinical trial to compare the cost- effectiveness of PAiT to standard 10 Hz rTMS as treatment for patients with TRD. The results of our study will offer professionals evidence from a sufficiently powered trial to determine whether the PAiT protocol is more effective than standard high-frequency rTMS. Specifically, it will assess whether PAiT leads to a shorter treatment duration for depression and greater societal or occupational participation among patients with TRD. In addition, this trial will provide further insights into the underlying mechanisms related to treatment effect, the effects of rTMS or accelerated intermittent theta-burst stimulation on cognitive domains such as executive functioning and emotion, possible differences in side effects, long-term effects, and factors contributing to possible relapse.

**Trial Registration:**

ClinicalTrials.gov NCT05900271; https://clinicaltrials.gov/study/NCT05900271

**International Registered Report Identifier (IRRID):**

DERR1-10.2196/70121

## Introduction

### Background

Novel therapies for depression are needed because more than 33% of patients with major depressive disorder are treatment resistant, that is, they do not respond sufficiently after treatment with 2 or more evidence‐based pharmacological or psychotherapeutic treatments [1]. Brain stimulation using repetitive transcranial magnetic stimulation (rTMS) or intermittent theta‐burst stimulation (iTBS), an alternative form of rTMS, has been shown effective with remission rates of 20% to 30% [2] in patients with treatment-resistant depression (TRD). rTMS and iTBS are noninvasive procedures, meaning that they do not involve surgery or general anesthesia. While the remission rates of rTMS and iTBS are lower than the 55% remission rate achieved by electroconvulsive therapy (ECT) [3], the most effective treatment for patients with depression, rTMS and iTBS have fewer side effects compared to ECT and antidepressant medication. Therefore, optimization of rTMS and iTBS therapies and their outcomes is a promising scientific goal.

### Novel Noninvasive Neuromodulatory Therapies

Transcranial magnetic stimulation (TMS), a noninvasive neuromodulatory therapy, has been added to the treatment arsenal for unipolar depression [4]. TMS is based on the principle of electromagnetic induction by applying magnetic pulses to the brain. This magnetic pulse induces an electrical field in the underlying neurons; when TMS pulses are applied repetitively, this effect outlasts the time of stimulation and leads to enduring effects on cortical excitability [5]. rTMS is recognized as a valuable therapeutic option in TRD [4]. Randomized controlled trials (RCTs) have consistently demonstrated the efficacy and safety of rTMS in TRD, with remission rates of approximately 25% [4,6-8]. In standard Food and Drug Administration–approved 10 Hz rTMS, 3000 pulses are given in 19 minutes; a train of 40 pulses lasting for 4 seconds is given every 15 seconds.

rTMS is not associated with common drug‐associated side effects (eg, nausea, sedation, akathisia, weight gain, and risk of intoxication) or the cognitive side effects often feared with ECT. The risk of developing side effects in response to rTMS is low [4,9]; transient headaches and localized scalp pain are the most common side effects and are self‐limiting or eminently treatable with paracetamol, acetaminophen, or other analgesics.

iTBS is a newer form of rTMS [10]. Magnetic pulses are applied in a certain pattern, called bursts. The basic element of theta-burst stimulation is a 3‐pulse burst at 50 Hz delivered every 200 ms (ie, 5 Hz). In the recently developed Stanford neuromodulation therapy (SNT) protocol, a train of 10 bursts lasting for 12 seconds is given every 10 seconds for 60 cycles in iTBS, resulting in a total session duration of 10 minutes. The main advantage of iTBS is that a similar number of pulses can be given in a shorter time frame [11,12]. This shorter time frame facilitates the administration of iTBS multiple times a day, contrary to the 24-hour spacing of standard 10 Hz rTMS [11,12]. Multiple daily iTBS sessions with adequate spacing of 50 minutes is likely to enhance the efficacy, as suggested by animal studies that showed hour‐long intervals may be optimal for producing long-term potentiation of neurons [13].

### Accelerated iTBS According to the SNT Protocol

There are 2 observational studies and 1 RCT on the SNT protocol (applying accelerated iTBS combined with functional magnetic resonance imaging [fMRI] task-based neuronavigation). The first observational study was a proof-of-principle study involving 6 participants [14]. The second study was a larger observational study [12]. The findings from a subsequent RCT resulted from the planned interim analysis [11]. These 3 studies reported an extremely high overall remission rate, at any point during the 4-week follow-up period, of 79% [15]. Dropout rates were low across all 3 studies; in the study by Williams et al [14], 1 of 5 patients dropped out due to lack of remission by day 4; in the observational study by Cole et al [12], 1 of 22 patients dropped out on day 1 of the trial due to a history of multiple therapeutic intolerances; and no participants dropped out in the RCT by Cole et al [11]. Overall, accelerated intermittent theta-burst stimulation (aiTBS) was well tolerated; reported side effects were similar to standard 10 Hz rTMS, that is, headache and scalp pain. No safety issues were reported. Although promising, it is unclear what the crucial element is of the SNT protocol: the intersession interval (ISI), accelerated delivery, high pulse dose, scalp-cortex correction, individualized targeting, or a combination of these factors.

The 50-minute spacing between sessions is in line with evidence from basic neuroscience research and human physiology data, showing that spaced iTBS sessions have an enhanced effect compared with the same number of single daily sessions [13]. Accelerated sessions, that is, more sessions per day, also contribute to nonlinear improvements in clinical symptoms. ISI of at least 50 minutes may enhance synaptic strengthening [12,13]. Finally, the functional connectivity–guided targeting method used in this study may have contributed to the high remission rate observed. The left dorsolateral prefrontal cortex (DLPFC) is a large brain area that consists of several subregions, some of which are correlated and some anticorrelated with subgenual anterior cingulate cortex (sgACC) activity. Recent studies suggest that these correlated and anticorrelated subregions are part of different affective circuits; stimulating a subregion of the left DLPFC that is anticorrelated with the sgACC reduces melancholic symptoms, resulting in lower depression scores [11,12]. This connection is not necessarily targeted with standard 10 Hz rTMS using the Beam-F3 method [16].

### Cost‐Effectiveness

No independent international studies exist that assess the cost‐effectiveness of SNT (as compared to standard rTMS) in the treatment of TRD. Given the patent restrictions on SNT, we sought to replicate SNT in our own personalized aiTBS protocol (personalized functional connectivity–guided aiTBS [PAiT]) to assess the cost-effectiveness of both treatments. Considering that treatment duration of the PAiT protocol is notably shorter compared to 10 Hz rTMS (1 week vs 6 weeks) and is expected to result in increased remission rates (ie, therefore obtaining more quality-adjusted life year benefits earlier), we will compare the 2 treatments on their cost-effectiveness to evaluate the potential added value of this innovative therapy for a more efficient health care system.

### Durability of the Effects of Personalized aiTBS

As of 2024, little was known about the long-term effects of personalized aiTBS. One could argue that the same remission or relapse course can be expected as for ECT, that is, quick antidepressant effects directly after the intensive 5-day treatment in combination with a high chance of relapse in the following weeks. Therefore, our study will have weekly clinical assessments for 7 weeks in both conditions to enable comparisons in treatment efficacy, during treatment, directly after treatment, and 1 week after treatment between rTMS and PAiT, along with follow-up clinical assessments at 6 and 25 weeks after treatment. In addition, we will investigate the (factors contributing to) side effects during the treatment in both conditions using an in-house questionnaire [17].

### Contribution to the Field of the Proposed Study

A well-powered and independent RCT is needed to determine the cost-effectiveness and safety of PAiT versus rTMS in patients with TRD.

## Methods

### Aim

The aim of the Dutch Depression Outcome Trial (D-DOTT) is to determine the cost‐effectiveness of PAiT versus standard daily 10 Hz rTMS in patients with TRD, who did not respond to 2 or more evidence‐based treatments, using an adequately powered RCT.

### Study Design

This multicenter single‐blind RCT consists of 2 phases in which participants will be randomly assigned to either standard 10 Hz rTMS (30 daily rTMS sessions in 6 weeks, providing a total of 90,000 magnetic pulses) or PAiT (50 iTBS sessions in 5 days, providing a total of 90,000 pulses) [11,12], with 4 follow‐up moments at 7, 12, 26, and 31 weeks after baseline. In either condition, the timing of these measurements translates to clinically relevant time points at 6 and 25 weeks after treatment, as well as aligned postbaseline follow-ups that are important for the cost-effectiveness analysis. As such, the follow-up measurement at 31 weeks after baseline is only used in the rTMS condition (25 weeks after treatment).

### Setting of the Study

**Figure 1:**
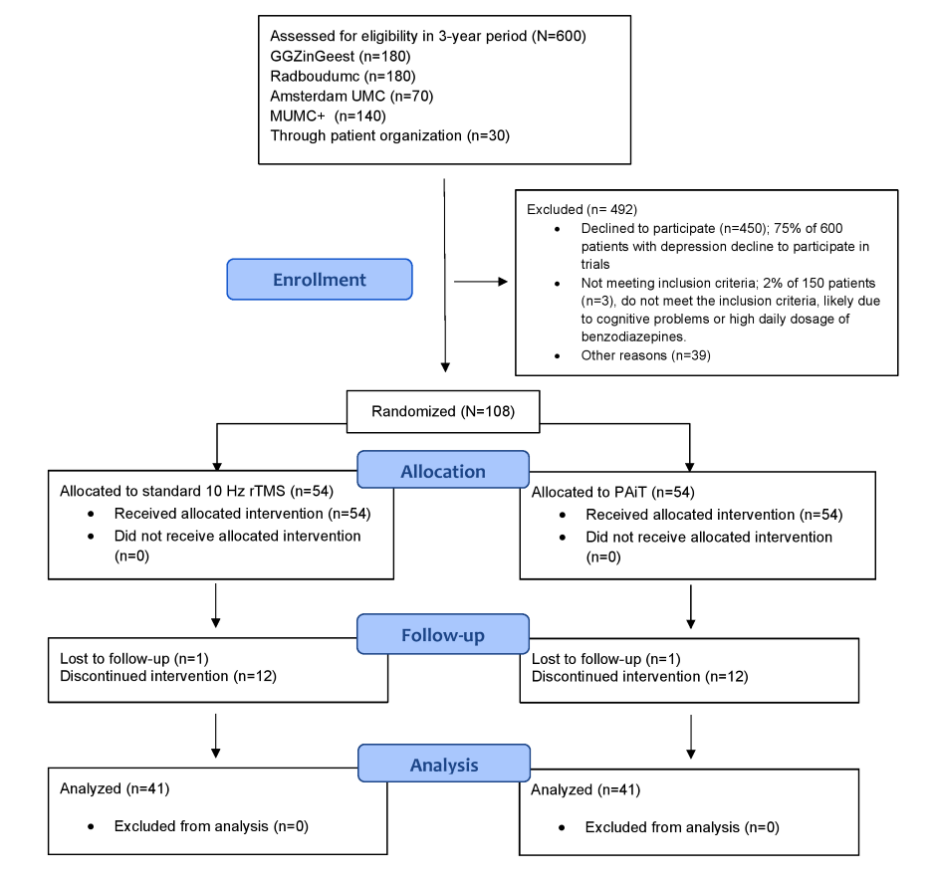
The CONSORT (Consolidated Standards of Reporting Trials) flowchart. UMC: University Medical Centre; MUMC: Maastricht University Medical Centre; rTMS: repetitive transcranial magnetic stimulation; PAiT: personalized functional connectivity–guided accelerated intermittent theta-burst stimulation.

This multicenter trial will take place within the Dutch specialized mental health care setting. Patients will be recruited from specialized outpatient clinics with ample experience in both the treatment of TRD and treatment with rTMS or iTBS, through self‐referral, and through the patient organization for depression (refer to the CONSORT [Consolidated Standards of Reporting Trials] flowchart in Figure 1)*.* Participating outpatient clinics are Radboudumc, GGZinGeest, Maastricht University Medical Centre+, and Amsterdam University Medical Centre. The following rTMS machines are used in this trial: MagStim Rapid2 (Amsterdam University Medical Centre, GGZinGeest, and Radboudumc), Neurosoft Neuro-MSX (Radboudumc), MAG and more powerMAG (GGZinGeest), and MagVenture MagPro X-100 (Maastricht University Medical Centre+). All sites use figure-of-8 coils. Localite is used for neuronavigation at all sites. The Neurosoft Neuro-MSX uses a built-in neuronavigation software.

### Participants

We aim to include 108 adult patients diagnosed with moderate to severe (Hamilton Depression Rating Scale >16) unipolar major depressive disorder without psychotic symptoms and who have had a failed response to at least 2 treatment trials with adequately dosed antidepressants or evidence-based forms of psychotherapy (interpersonal therapy, cognitive behavioral analysis system of psychotherapy, behavioral therapy, cognitive behavioral therapy, cognitive bibliotherapy, problem‐solving therapy, brief psychodynamic therapy, and reminiscence therapy). Exclusion criteria include lifetime diagnosis of bipolar disorder, current psychotic disorder, substance abuse disorder, suspected dementia, active suicidal thoughts with intent to act on them, patients with epilepsy, patients with large or ferromagnetic metal parts in the head (except for a dental wire), implanted cardiac pace maker or neurostimulator, or inability to understand or comply with study requirements.

These exclusion criteria serve 2 purposes: first, to ensure a clinically homogeneous sample of patients with unipolar depression, free from comorbid conditions that could affect treatment effectiveness; and second, to comply with safety guidelines related to rTMS, the accelerated iTBS protocol, and the required magnetic resonance imaging (MRI) procedures, as well as general safety standards.

### Study Procedures

Study information is provided to eligible participants, and these patients are contacted again after at least 7 days to discuss participation and any remaining questions. If patients choose to participate, they will be invited to a research intake session, during which they will sign the informed consent form and undergo screening for inclusion and exclusion criteria. Participation is voluntary, and individuals may withdraw from the study at any time. Table 1 shows an overview of the study. Participants who were allocated to standard 10 Hz rTMS will be offered PAiT after the end of the study (eg, 25 weeks after the last rTMS session). We will request patients allocated to PAiT to postpone enrolling in standard 10 Hz rTMS for 25 weeks after the last treatment session. This is merely a request as patients are allowed to ask for treatment as usual, including standard 10 Hz rTMS. Our participating treatment centers deem it not ethical to withhold this usual 10 Hz rTMS treatment upon a patient’s request. In addition to clinical effectiveness, we will study patient‐reported outcomes, tolerability, and safety. Alongside this RCT, we will also conduct an economic evaluation and evaluate facilitating and hindering factors related to implementation in the group of researchers and health care professionals involved in this study. The funding agency, the Efficiency Studies arm of the Care Research Netherlands and Medical Sciences of the Dutch Research Council, has explicitly requested that the trial be based in care as usual.

**Table 1:** An overview of the study for both treatment conditions.

			rTMS^a^		PAiT^b^
**T0**					
	Week –2	Screening: clinicometrics; biobank; MRIc pre+additional tasks; rTMS premeasurements		Screening: clinicometrics; biobank; MRI pre+additional tasks; PAiT premeasurements	
**T1**					
	Week 0	Baseline		Baseline	
**T2**					
	Week 1	Treatment Clinicometrics (weekly)		Treatment Clinicometrics (daily)	
	Week 2	Biobank		Follow-up (1 week): clinicometrics (weekly); MRI post+additional tasks; biobank	
	Week 6	Clinicometrics		Clinicometrics	
**T3**					

	Week 7	Follow-up: clinicometrics; MRI post+additional tasks; biobank		Follow-up: clinicometrics; biobank	
**T4**					
	Week 12	Follow-up: clinicometrics; biobank		Follow-up: clinicometrics	
**T5**					
	Week 26	Follow-up: clinicometrics		Follow-up (25 weeks): clinicometrics	
**T6**					
	Week 31	Follow-up (25 weeks): clinicometrics		Clinicometrics	

^a^rTMS: repetitive transcranial magnetic stimulation.

^b^PAiT: personalized functional connectivity–guided accelerated intermittent theta-burst stimulation.

^c^MRI: magnetic resonance imaging.

### Randomization

Participants will be randomly assigned to either the rTMS or the PAiT arm. Randomization will be done via Castor EDC and will be stratified for treatment center. We will use block randomization using random sequences of block sizes of 2, 4, and 6. This approach ensures treatment assignment balance within each complete block and minimizes the imbalance of group sizes within clinical centers, thus correcting for confounding from possible differences between treatment sites (such as differences in experience with rTMS). In addition, before the start of the trial, we will note patient’s preferred treatment, that is, standard 10 Hz rTMS or PAiT. The expected study duration is 48 months, and inclusion of patients started in February 2024.

### Intervention

The PAiT protocol, modeled after the SNT protocol [12], consists of 5 days of treatment in which a total of 90,000 magnetic pulses are given over 50 sessions (10 sessions per day, 50 minutes ISI). iTBS at 90% of the resting motor threshold corrected for cortical depth will be administered to the location within the left DLPFC showing the strongest anticorrelation with the sgACC. This is achieved using participant‐specific, resting state–fMRI‐guided neuronavigation. The coordinates within the DLPFC most anticorrelated with the sgACC are identified using the seedmap- and cluster-based method described by Cash et al [18]. The cluster threshold is set to 2.5%, meaning 2.5% of the most anticorrelated voxels are retained in the DLPFC before clustering. The center of gravity of the largest cluster is defined as the target coordinate. The target coordinate and corresponding stimulation site are located for each individual using neuronavigation software. The resting motor threshold is corrected for cortical depth at the stimulation site using the formula described by Stokes et al [19]. The BrainRuler software [20] is used to determine the cortical distance.

### Comparator

The comparator consists of 6 weeks of standard rTMS treatment, following the Dutch depression treatment guidelines [21]. This involves delivering a total of 90,000 magnetic pulses across 30 sessions (1 session per day), using 10 Hz stimulation at 120% of the resting motor threshold. Stimulation is applied to the left DLPFC, localized using the Beam-F3 method [16,22], which identifies the DLPFC based on standardized skull measurements.

### Image Acquisition and Preprocessing

Whole brain structural and functional images are acquired within 2 weeks before treatment and within 2 weeks after treatment. Scans will be performed on a 3-T whole-body scanner with standardized and optimized sequences. Sequences included in the pre- and posttreatment scans are described in Textbox 1.

Preprocessing of the functional and structural images is performed using the Freesurfer software suite, fMRIprep, an MRI quality control tool, and the FMRIB software library suite. Functional images are denoised and smoothed for stimulation target identification. Denoising was performed using global signal regression, 6 motion correction parameters, 8 physiological regressors, and spike removal. Smoothing was applied with a 6-mm full width at half maximum kernel.

**Sequences included in the pre- and posttreatment scans.**

Pretreatment scans

- T1-weighted structural MRI (5 min): repetition time (TR)=2300 ms, echo time (TE)=2.26 ms, 192 slices, slice thickness=1.0 mm, voxel size=1×1×1 mm, and flip angle=8°
- Resting state–functional MRI (2×10 min): TR=1200 ms, TE=30 ms, 64 slices, slice thickness=2.0 mm, 488 volumes, voxel size=2×2×2 mm, Simultaneous Multi-Slice (SMS) factor=4, and flip angle=66°
- Diffusion tensor imaging (8 min): TR=3100 ms, TE=102 ms, 66 slices, slice thickness=2.0 mm, voxel size=2×2×2 mm, and SMS factor=3
- Tower of London task functional MRI (5.5 min): TR=1200 ms, TE=30 ms, 64 slices, slice thickness=2.0 mm, 488 volumes, voxel size=2×2×2 mm, SMS factor=4, and flip angle=66°
- Hariri Emotion Recognition task functional MRI (5.5 min) [23]: TR=1200 ms, TE=30 ms, 64 slices, slice thickness=2.0 mm, 488 volumes, voxel size=2×2×2 mm, SMS factor=4, and flip angle=66°
- Total scanning time=44 minutes

Posttreatment scans

- T1-weighted structural MRI (5 min): TR=2300 ms, TE=2.26 ms, 192 slices, slice thickness=1.0 mm, voxel size=1×1×1 mm, and flip angle=8°
- Resting state–functional MRI (1×10 min): TR=1200 ms, TE=30 ms, 64 slices, slice thickness=2.0 mm, 488 volumes, voxel size=2×2×2 mm, SMS factor=4, and flip angle=66°
- T2-weighted fluid attenuated inversion recovery structural MRI (6 min): TR=5000 ms, TE=386 ms, inversion time=1650 ms 176 slices, slice thickness=1.0 mm, and voxel size=1×1×1 mm
- Diffusion tensor imaging (8 min): TR=3100 ms, TE=102 ms, 66 slices, slice thickness=2.0 mm, voxel size=2×2×2 mm, and SMS factor=3
- Tower of London task functional MRI (5.5 min): TR=1200 ms, TE=30 ms, 64 slices, slice thickness=2.0 mm, 488 volumes, voxel size=2×2×2 mm, SMS factor=4, and flip angle=66°
- Hariri Emotion Recognition task functional MRI (5.5 min): TR=1200 ms, TE=30 ms, 64 slices, slice thickness=2.0 mm, 488 volumes, voxel size=2×2×2 mm, SMS factor=4, and flip angle=66°
- Total scanning time=40 minutes

### Outcome Measures

#### Main Study Parameter or End Point

Remission of depression 1 week after the last treatment session was defined as a score of ≤7 on the 17-item Hamilton Depression Rating Scale (HDRS‐17).

#### Secondary Outcomes

In addition to the primary outcomes, secondary outcomes will be assessed to provide a more comprehensive understanding of the effectiveness and impact of rTMS/PAiT. First, an economic evaluation based on the general principles of a cost‐effectiveness (utility) analysis, which will be performed alongside the intervention by comparing patients treated with PAiT with those receiving standard 10 Hz rTMS. In addition, societal costs and health consumption will be assessed for the economic evaluation at baseline and 7, 12, and 26 weeks after baseline. We will use health‐related quality of life determined with the EQ‐5D‐5L and health care use or costs, patients’ and their family’s out‐of‐pocket costs, and productivity losses owing to absenteeism measured with the Trimbos Institute and Institute for Medical Technology Assessment Cost questionnaire for Psychiatry [24]. Second, patient‐reported outcome measures will be recorded, that is, positive mental health, as measured with Mental Health Continuum‐Short Form [25] and subjective memory loss. Third, percentage reduction of depressive symptoms will be measured with self‐rated 9-item Patient Health Questionnaire (PHQ-9) on each treatment day and the HDRS‐17. Fourth, relapse will be measured at 6 and 25 weeks after treatment. Relapse was defined as an HDRS‐17 total score of ≥15 for 2 consecutive assessments separated by 5 to 15 days or hospitalization for depression. Fifth, we establish tolerability of the treatment and side effects of PAiT and rTMS [9]. Sixth, we will identify health care system–, clinician‐, and patient‐related factors that influence the implementation of PAiT were identified to inform the development of an implementation plan. Seventh, we will compare patient-reported changes in mood 1 week after treatment (as measured with self-rated PHQ-9) with clinician-reported clinical impression (as measured with Clinical Global Impression [23]).

#### Exploratory Outcome Measures

In addition, we want to untangle the effects of possible biomarkers and predict treatment outcomes, including cardiovascular risk factors (waist:hip ratio, ankle arm index [a measure to determine peripheral atherosclerosis], c-reactive protein, triglyceride, high-density lipoprotein cholesterol, total cholesterol, and glycated hemoglobin levels); neurophysiological biomarkers, that is, using heart rate reduction, heart brain coupling, and an increase in heart rate variability; magnitude and angle of the electric field induced by rTMS in the cortex; structural and functional brain networks as biomarkers for clinical response; effects of cortical depth; and structural and functional brain network changes induced by 10 Hz rTMS and PAiT (and their relation to treatment outcome), as measured by structural and functional MRI.

We also aim to assess changes in emotional and cognitive task performance (Hariri [26,27] and Tower of London [28]) and task-related brain activation induced by 10 Hz rTMS and PAiT (and their relation to treatment outcome), as measured by task-based functional MRI; assess the changes in Pavlovian bias induced by 10 Hz rTMS and PAiT (and their relation to treatment outcome), as measured by a behavioral controllability task (RobotMadness, refer to Dorfman et al [29] for more information regarding controllability and Pavlovian biases); and to establish a biobank to determine standard 10 Hz rTMS– and PAiT-induced changes, as measured with blood derived biomarkers. Markers of interest include brain-derived neurotrophic factor and epigenetic changes for rTMS-induced plasticity, genotyping for treatment response prediction.

### Blinding

Participants and those who will be administering the rTMS or PAiT treatment cannot be blinded. Those carrying out clinical assessments will be blinded. Blind rating will be carried out via video-recorded interviews with participants. Interviewers will not be blinded to the treatment conditions and, therefore, will be thoroughly instructed to carry out the interview in a structured and objective manner. A blinded rater will score the videotaped clinical assessments. To ensure minimal bias effects, 20% (227/1134) of interviews will be randomly assessed on interrater reliability and interview quality. All video footage will be deleted after rating the interviews and after the independent monitor has reviewed and agreed. The clinical ratings will be stored for further analysis.

### Sample Size Calculations

Our trial should consist of 108 patients (for a 2‐sided test at α=.05 and with a power=0.80). Remission rates of patients treated with SNT in 2 open‐label studies and 1 interim analysis from an RCT were extremely high, that is, 79%. The remission rates in patients treated with ECT, the most well-known and effective treatment for TRD, is approximately 55% (range 48%‐65%). Our sample size calculation is based on the conservative assumption that our PAiT protocol is at maximum as effective as ECT in patients with TRD. For our sample size calculation, we used the 55% remission rate and the known remission rate for standard 10 Hz rTMS, 25%. We will oversample the necessary 82 patients with 32% to adequately handle possible attrition, as the new intervention will be relatively intense compared to standard 10 Hz rTMS treatment, resulting in a required sample size of 108 patients.

### Statistical Analyses

#### Overview

Statistical analyses will be performed on the intention‐to‐treat as well as the per protocol sample. Missing data will be replaced using the multiple imputation method. For all analyses, corrected *P* values of <.05 will be regarded as statistically significant. Safety data (eg, side effects) will be collected and summarized in the study results. A description of the analyses for the primary, secondary, and exploratory objectives is provided in the following sections.

#### Primary Study Parameters

For the intention‐to‐treat analysis, we will use a chi-square test to determine differences in remission 1 week after treatment.

#### Secondary Study Parameters

The economic evaluations, that is, cost‐utility analysis, will be conducted in accordance with the Dutch guidelines for economic evaluations. The outcome measure for the cost‐utility analysis will be quality-adjusted life years, measured using the Dutch tariffs, that is, utility weights of the EQ‐5D‐5L [30-32]. Total costs will be estimated using a bottom‐up (or microcosting) approach, where information on each element of service used is multiplied by an appropriate unit cost and summed to provide an overall total cost. We will consider four types of costs: (1) intervention costs, (2) costs stemming from health care uptake, (3) patients’ and their family’s out‐of‐pocket costs, and (4) costs stemming from productivity losses due to absenteeism. The recall period for all resource use measurements will be 3 months. A baseline analysis will be performed to examine the comparability of groups at baseline in terms of costs and outcomes. If necessary, the methods will be applied to control for differences in baseline [33]. The incremental cost‐effectiveness ratio (ICER) will be computed to obtain the incremental costs per quality-adjusted life years gained. Stochastic uncertainty will be handled using 5000 nonparametric bootstraps and by plotting the simulated ICERs on the ICER plane. The bootstrap replications will be used to calculate the 95% CIs for the costs based on the 2.5th and 97.5th percentiles. For decision-making purposes, the ICER acceptability curve will be plotted for various willingness‐to‐pay ceilings. Missing data will be handled using single imputations nested in the nonparametric bootstrap samples [34].

For the patient‐reported outcome measures, that is, positive mental health, as measured with Mental Health Continuum‐Short Form [25]. We will use a linear mixed model on the data of the 3 measurements (baseline, directly after last treatment, and 26 weeks after baseline), comparing differences in the treatment effect. In addition, in order to assess subjective memory complaints, the Subjective assessment of memory impairment (subjective memory) assessment will be done at baseline, after the last treatment, and 26 weeks after baseline.

For the Percentual reduction of depressive symptoms, as measured with self‐rated PHQ-9 on each day using the PAiT protocol, compared to weekly reduction of depressive symptoms, as measured with PHQ-9 during standard 10 Hz rTMS we will use linear mixed models. Similarly, we will determine percent reduction of depressive symptoms, as measured with the clinically assessed HDRS‐17 before, during, and after treatment, as well as at the follow-up moments.

Relapse, at 6 and 25 weeks after treatment, defined as (1) sustained remission, that is, HDRS-17≤7, and (2) sustained response, that is, response at end of treatment maintained through week 6 and 25. During treatment phase (T2), we will conduct the HDRS-17 weekly for 6 weeks for both treatment arms. By doing this, using the chi-square test, we can compare the remission rate with PAiT after 6 weeks to 6 weeks of treatment with standard 10 Hz rTMS; this is of interest as patients allocated to PAiT only receive treatment for 1 week.

We will report the prevalence of side effects during and directly after treatment based on international recommendation [17]. We will establish the tolerability and side effects of PAiT and rTMS. We will use qualitative data collected while supporting the implementation of PAiT, to analyze health care system–, patient‐, and clinician‐related factors that influence the implementation of PAiT in clinical settings. We will use a 1-tailed paired *t* test or an ANOVA to compare patient-reported changes in mood 1 week after treatment (as measured with self-rated PHQ-9) with clinician-reported clinical impression (as measured with the Clinical Global Impression scale).

### Ethical Considerations

This clinical trial was approved by the institutional ethics board of Amsterdam University Medical Centre in August 2023 (2023.0437—NL83892.018.023) and was registered at clinicaltrials.gov (NCT05900271) on June 12, 2023. All participants will provide verbal and written informed consent before completion of any study measures. Data will be deidentified and, after completion of data collection, transmitted to a secure repository with safeguards in place to protect patients confidentiality.

### Data Monitoring

Each clinical site will manage the internal quality of study conduct, data collection, documentation, and completion. All sites will adhere to a unified quality management plan. Before starting data collection, the principal investigators and the independent monitor confirmed that the necessary safety measures were in place at each site. The principal investigators convened with the full research team to review the study protocols, focusing on the definition of outcomes, study design, procedures for recording and reporting adverse events, informed consent procedures, and documentation. Staff will be trained to conduct assessments, ensuring they understand data collection procedures, processes, and the requirements for reporting adverse events.

## Results

Recruitment for this RCT started in February 2024. As of December 2024, we had enrolled 31 patients; the last participant is expected to complete their posttreatment assessments in January 2027. Publication of the study’s findings is anticipated around mid-2027.

## Discussion

### Importance and Broader Impact of the Study

This study protocol describes an adequately powered multicenter RCT investigating the cost-effectiveness of our PAiT protocol compared to standard 10 Hz rTMS in patients with TRD who did not respond to 2 or more evidence‐based treatments. Our proposed RCT is important because the limited number of studies using the original personalized aiTBS protocol from the Stanford group (SNT) were performed in relatively small samples. Our trial can provide insight into the clinical effectiveness of the PAiT protocol and its cost‐effectiveness. In addition to the clinical trial, primarily aimed at cost-effectiveness, our study also includes a number of exploratory neurophysiological outcome measures, which help in elucidating underlying mechanisms of treatment and eventually help in the identification and development of biomarkers that may guide treatment beyond clinical measures.

### Principal Findings

It is hypothesized that the PAiT protocol will be more cost-effective than standard 10 Hz rTMS in patients with TRD, achieving a remission rate of 55%, comparable to treatment with ECT. We believe that our PAiT protocol may not be as effective as the original SNT protocol, with an apparent efficacy of 79% [11], due to methodological differences in the primary outcome measurement (remission at 1 week after treatment vs remission at any time point during the 4-week follow-up period) and the anticipated higher treatment resistance rate in our patient population. Remission rates immediately after aiTBS treatment have been seen to vary widely between 10% and 86.4% [15], possibly due to differences in the treatment protocol (sessions per day and amount of treatment days) and patient characteristics. Furthermore, the results of our study will be compared with those of other ongoing trials investigating the efficacy of aiTBS.

Given its notably shorter treatment duration (1 week vs 6 weeks) and the expected increased remission rates, quality-adjusted life years gains will be obtained earlier with similar treatment costs. In addition, PAiT is expected to increase societal or occupational participation of the patients, leading to reduced health care costs and productivity in the long term. Therefore, PAiT is anticipated to be more cost-effective compared to rTMS.

### Challenges of the Proposed Trial

We anticipate that patients are drawn toward our trial because the 1-week intensive treatment is appealing to them. However, these patients may not be willing to be randomized. We aim to tackle this by offering PAiT after the end of the study participation (eg, 25 weeks after the last rTMS session) to patients who are randomized to the 6-week rTMS arm. To date, the PAiT protocol, modeled after the SNT protocol for TRD, is only offered in the Netherlands in our trial.

Another challenge is that the PAiT protocol with 10 sessions on 5 consecutive days is intense, and it is anticipated that patients may miss sessions due to various reasons (eg, traffic or oversleeping). In a complete course, participants can miss 20% (10/50) of sessions at maximum. If >20% (10/50) of sessions are missed, it is considered a failed course. In the rTMS condition, depending on feasibility for the patient and the rTMS team, a maximum of 6 missed sessions may be added to the end of the course. In the PAiT condition, in concordance with the Stanford group, missed sessions will not be rescheduled. If optimal adherence to the protocol is not possible for a patient due to limited traveling capacity or due to the distance from the treatment center, we offer admission to the ward.

To further facilitate tolerability of the PAiT condition, separate rooms will be provided for the patients to rest in between sessions.

This clinical trial includes a direct comparison between treatments of varying durations. This poses the challenge of establishing a representative time point for measuring the primary outcome. To capture the clinical effects in more detail and over time, we will perform weekly clinical measurements for 7 weeks following the start of the treatment regardless of the treatment condition. The time point of measuring the primary outcome was set to 7 days following the final treatment session.

### Relevance for Practice

PAiT can be applied in various treatment settings and by various health care professionals. If positive, the results of our study will provide Dutch and international professionals with an adequately powered trial answering whether PAiT is an evidence‐based therapy for depression. A novel therapy is necessary to overcome TRD in patients who did not respond after 2 or more evidence‐based treatments. In addition, this trial will provide further insights into the differences in the side effects, long-term effects, and (factors contributing to) possible relapse between rTMS and PAiT.

Different rTMS equipment will be used at different sites in this trial. Technical and practical differences in the equipment may introduce variability across sites. However, using different machines is important for generalizability of the results because, in daily practice, mental health institutions use different rTMS machines. To minimize variability in the practical application of rTMS, we thoroughly instruct technicians to follow our provided training and standard operating procedures. All treatments will be conducted using neuronavigation systems with a calibration accuracy of less than 3 mm. In addition, we will control for rTMS machine or site to ensure the reliability of our analyses and investigate the impact of different equipment.

### Patient and Public Involvement

To contribute to meaningful patient and public involvement in research [35], an individual with lived experience, affiliated with the Dutch Depression Association (Depressie Vereniging), has been actively engaged since the development of the study protocol and the submission of the funding application for the D-DOTT trial. This collaboration serves to ensure that the research remains responsive to, and reflective of, the priorities and perspectives of individuals affected by depression.

### Dissemination Plan

The findings from this clinical trial will be disseminated through a peer-reviewed journal article, presentations at academic conferences, and promotion via social media.

## Appendix

Multimedia Appendix 1 Grant award document from ZonMW (the Netherlands Organisation for Health Research and Development).

Multimedia Appendix 2 Peer review comments from DoelmatigheidsOnderzoek, ZonMw program, Netherlands Organisation for Health Research and Development.

## Abbreviations

aiTBS: accelerated intermittent theta-burst stimulation
DLPFC: dorsolateral prefrontal cortex
ECT: electroconvulsive therapy
fMRI: functional magnetic resonance imaging
HDRS-17: 17-item Hamilton Depression Rating Scale
ICER: incremental cost‐effectiveness ratio
ISI: intersession interval
iTBS: intermittent theta‐burst stimulation
MRI: magnetic resonance imaging
PAiT: personalized functional connectivity–guided accelerated intermittent theta-burst stimulation
PHQ-9: 9-item Patient Health Questionnaire
RCT: randomized controlled trial
rTMS: repetitive transcranial magnetic stimulation
sgACC: subgenual anterior cingulate cortex
SMS: Simultaneous Multi-Slice
SNT: Stanford neuromodulation therapy
TE: echo time
TR: repetition time
TRD: treatment-resistant depression

## Data Availability

Data sharing is not applicable to this article as no data sets were generated or analyzed during this study.
