# Supplementary material for: Comparison of 5-Day Multidaily Neuronavigated Theta-Burst Sessions With 6-Week Standard Repetitive Transcranial Magnetic Stimulation (the Dutch Depression Outcome Trial): Protocol for a Randomized Controlled Trial
Source: JMIR Res Protoc. 2025 Aug 21;14:e70121. doi: 10.2196/70121 (PMC12411794; doi:10.2196/70121)
Supplement: Multimedia Appendix 1 [file resprot_v14i1e70121_app1.pdf]

Nederlandse organisatie voor gezondheidsonderzoek en zorginnovatie

Laan van Nieuw Oost-Indië 334
2593 CE Den Haag
Postbus 93245
2509 AE Den Haag
Telefoon 070 349 51 11
Fax 070 349 51 00
www.zonmw.nl
info@zonmw.nl

Amsterdam UMC
Raad van Bestuur
De heer C.H. Polman
Boelelaan 1117
1081 HV Amsterdam

Dossiernummer
10390012110079

Datum
7 juli 2022

Contactpersoon
Laurien Hoefnagel
Telefoon 070 349 5465
doelmatigheidsonderzoek@zonmw.nl

Onderwerp
Subsidiebesluit over dossiernummer 10390012110079

Geachte heer Polman,

Met veel genoegen informeren wij u dat het bestuur van ZonMw besluit uw subsidieaanvraag, *Higher, faster, better: a study on (cost-) effectiveness of neuromodulation in depression. Is an accelerated intermittent Theta Burst Stimulation protocol compared to standard 10 Hz repetitive Transcranial Magnetic Stimulation, more (cost-) effective in patients with treatment-resistant depression?*, te honoreren. U leest in deze brief meer over de onderbouwing van het besluit en de verplichtingen waaraan u moet voldoen voordat uw project kan starten. In bijlage 1 leest u de aanvullende verplichtingen die voor u als subsidieontvanger gelden.

Subsidiebedrag en duur project
U ontvangt een subsidie van maximaal € 740.550,- voor de duur van maximaal 48 maanden, ten opzichte van uw ingediende begroting aangepast op de volgende punten; de kosten voor METC zijn gemaximeerd tot € 2.000,-. De correctie bedraagt € 4.000,-. De benchfee is gemaximeerd op basis van het percentage FTE (Phd 100%; Postdoc 56%) op € 7.800,-. De correctie bedraagt € 200,-. De zorgkosten (50% van de post Neuronavigation) dienen, zoals aangegeven op de begroting, uit de eigen bijdrage voldaan te worden. Van het subsidiebedrag is € 5.000,- gereserveerd voor open access publicatie kosten. Het subsidiebedrag is inclusief eventuele btw.

Van de verschillende posten dient bij de eindafrekening een specificatie te worden aangeleverd.

Onderbouwing besluit
Alle subsidieaanvragen zijn beoordeeld op relevantie en kwaliteit zoals vermeld in de subsidieoproep Open ronde 2023, onderzoek naar de effectiviteit en kosten van interventies. De programmacommissie Evaluatie van Effecten en Kosten (EEK) van het programma DoelmatigheidsOnderzoek adviseert positief over uw subsidieaanvraag. Het advies van de commissie is naar het oordeel van ZonMw op zorgvuldige wijze tot stand gekomen. ZonMw heeft dit advies overgenomen en haar besluit hierop gebaseerd.

Oordeel over relevantie
De commissie heeft het volgende oordeel over de relevantie van uw subsidieaanvraag: **zeer relevant**. Het oordeel van het panel van Patiëntenfederatie Nederland is hierin meegewogen.

Het relevantieoordeel is op de volgende argumenten gebaseerd:

- De commissie merkt op dat de veiligheid en werkzaamheid van Transcraniële Magnetische Stimulatie (TMS) in diverse studies is bevestigd. Verder zal voorafgaand aan de behandeling een MRI uitgevoerd worden om macroscopische hersenafwijkingen uit te kunnen sluiten. De commissie adviseert daarnaast een interim analyse uit te voeren en een Data Safety Montitoring Board (DSMB) in te stellen om de veiligheid te bewaken.
- In het project wilt u onderzoeken of MRI-gestuurde intermitterende Theta Burst Stimulatie effectiever is dan standaard repetitieve transcraniële magnetische stimulatie (rTMS) als behandeling bij patiënten met een therapieresistente depressie. U verwacht een stijging van de effectiviteit van 25% met de standaardbehandeling naar 55% met het SNT protocol. De commissie is van mening dat er hierdoor substantiële kostenreductie en gezondheidswinst te behalen valt voor een grote groep patiënten.
- De vraagstelling van uw onderzoek sluit aan bij één van de prioriteiten op de kennisagenda van de Nederlandse Vereniging voor psychiatrie. De commissie is van mening dat de interventie bij een positieve uitkomst van de studie een verandering in het veld teweeg kan brengen en dat de studie kan bijdragen aan een aanpassing van de richtlijn.
- ZonMw hecht veel waarde aan patiëntenparticipatie en wil het patiëntenperspectief terugzien in uw subsidieaanvraag en onderzoek. De commissie is met het panel van de Patiëntenfederatie Nederland van mening dat relevant onderzoek betreft. Daarnaast zijn er goede aanpassingen gemaakt naar aanleiding van opmerkingen van het panel.

Oordeel over kwaliteit
De commissie heeft het volgende oordeel over de kwaliteit van uw subsidieaanvraag: **zeer goed**. Dit oordeel is gebaseerd op uw subsidieaanvraag, de beoordeling door referenten en het wederhoor.

Het kwaliteitsoordeel is op de volgende argumenten gebaseerd:

- Referent 3635953 merkt op dat alhoewel het uiterst moeilijk is adequate blindering van patiënten in dit type studie toe te passen, het ontbreken hiervan een zwaktepunt van de studie is. Om valide beoordelingen van uitkomstmaten te garanderen, zult u in de studie geblindeerde beoordelaars gebruiken. Bovendien zullen patiënten hun eigen remissie bepalen door een zelfbeoordelingsvragenlijst te gebruiken om verandering in depressieve symptomen te monitoren. Op verzoek van referent R3708901 gaat u in het wederhoor uitgebreider in op de blindering voor de beoordelaars.
  De commissie is van mening dat gegeven het feit dat patiënten niet geblindeerd kunnen worden er het maximale aan is gedaan om bias te voorkomen.
- Referent 3635953 geeft daarnaast aan dat ‘The Last Observation Carry Forward approach” bij ontbrekende data in het data-analyse plan outdated is. In het wederhoor komt u terug op dit punt. De ontbrekende data zullen feitelijk het ontbreken van metingen na de interventie zijn als gevolg van de uitval van patiënten uit de studie. U wilt het effect van de interventie onderzoeken onder de Missing At Random (MAR) assumptie, waarbij het erop neer komt dat u ervan uitgaat dat de uitval niet selectief is. Om de

impact van deze assumptie te bestuderen zal u een sensitiviteitsanalyse doen.

- De commissie vindt dat het plan van aanpak adequaat is en de onduidelijkheden zijn opgelost met het wederhoor. Zij merkt op dat veel suggesties van de referenten goed worden overgenomen zoals het toevoegen van response en suïcide ideatie als uitkomstmaat aan de studie. Ook is het nu mogelijk dat patiënten die niet responderen op de vergelijkende behandeling na afloop van de trial kunnen overstappen naar de andere interventie.
- Zowel de referenten als de commissie zijn er van overtuigd dat de projectgroep beschikt over de juiste expertise en ervaring om de studie succesvol uit te voeren

Samenvattend oordeel
Op basis van het eindoordeel over de relevantie en kwaliteit van uw subsidieaanvraag kan uw aanvraag gehonoreerd worden. In totaal zijn voor deze subsidieronde 39 aanvragen ingediend, waarvan we er 12 hebben gehonoreerd.

Start project
Voordat uw project kan starten moet u aan onderstaande verplichtingen voldoen.

Binnen **4 weken** na de verzenddatum van deze brief:

- Dient u het meldingsformulier in te dienen, hiervoor staat een taak klaar in Mijn ZonMw.

Samenwerking en bijdragen van derden
Stuur binnen **2 maanden** na de verzenddatum van deze brief een finale conceptversie (goedgekeurd door partijen maar nog niet ondertekend) van de samenwerkingsovereenkomst per e-mail naar ZonMw. Als ZonMw de betreffende overeenkomst niet accepteert, kan ZonMw besluiten dat de subsidie niet wordt verleend. Een kopie van de door alle partijen ondertekende versie van de samenwerkingsovereenkomst moet voor de startdatum van het project door ons zijn ontvangen. In de bijlage en op de ZonMw-webpagina Subsidies en Samenwerking/bijdragen van derden vindt u meer informatie.

Open science - Datamanagement
Om data uit uw project ook in de toekomst herbruikbaar te laten zijn, moet u een datamanagementplan opstellen. Informatie over hoe u dat moet doen, vindt u op de ZonMw-website. Volg vanaf deze link de stappen in de procedure.
Dien uiterlijk **3 maanden** na de verzenddatum van deze brief uw datamanagementplan en (voorlopige) kerngegevens in. Beide kunt u sturen naar doelmatigheidsonderzoek@zonmw.nl. Als u geen dataverzameling opbouwt, meldt u dat bij het programmateam.

Verklaringen en vergunningen
Voor de start van uw project moet u aan alle eisen voor het uitvoeren van het onderzoek hebben voldaan. Wij raden u aan eventuele procedures hiervoor tijdig te starten. Denk bijvoorbeeld aan een positief oordeel van een erkende medisch-ethische toetsingscommissie (METC), de Centrale Commissie Mensgebonden Onderzoek (CCMO), een projectvergunning van de Centrale Commissie Dierproeven (CCD), of een vergunning krachtens de Wet op het Bevolkingsonderzoek (WBO). U kunt bij de betreffende instanties nagaan of uw project dergelijke verklaringen of vergunningen nodig heeft.

Eerste voorschot
Als u aan alle verplichtingen voor de start van uw project heeft voldaan ontvangt u bericht over de bevoorschotting van uw project en ontvangt u het eerste voorschot.

Vragen
Heeft u vragen? Benader dan uw contactpersoon: Laurien Hoefnagel. Dat kan via e-mail: doelmatigheidsonderzoek@zonmw.nl of via telefoonnummer: 070 349 5465. Houd uw dossiernummer bij de hand zodat wij u snel kunnen helpen. Uw dossiernummer is: 10390012110079.

Bezwaar of klacht?
Bent u het niet eens met dit besluit? Dan heeft u de mogelijkheid om een bezwaarschrift in te dienen. Mocht u dat overwegen, dan adviseren wij u eerst uw contactpersoon te benaderen. Een bezwaarschrift moet u binnen **6 weken** na de verzenddatum van deze brief sturen naar het bestuur van ZonMw, t.a.v. Commissie Bezwaarschriften ZonMw, Postbus 93 245, 2509 AE Den Haag.

Als u ontevreden bent over de wijze waarop ZonMw uw aanvraag heeft behandeld, kunt u ons dit laten weten of een klacht indienen. Meer informatie vindt u op de ZonMw-website via
www.zonmw.nl/signalerenklagenbezwaarmaken.

Ik feliciteer u met de honorering van uw subsidieaanvraag en wens u succes bij de uitvoering van uw project!

Met vriendelijke groet,
namens het bestuur van ZonMw,

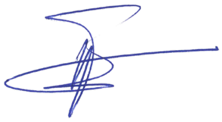

Veronique Timmerhuis
Algemeen directeur

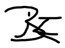

Kopie wordt per mail verzonden aan:
Mevrouw A. Dols
De heer E. van Exel

Bijlage – Aanvullende verplichtingen subsidieontvanger

Op dit besluit zijn de Algemene Wet Bestuursrecht en de Algemene subsidiebepalingen ZonMw van toepassing. Naast de voorwaarden uit de Algemene subsidiebepalingen ZonMw zijn onderstaande aanvullende verplichtingen van toepassing op uw subsidie. Aan deze verplichtingen moet u voldoen tijdens en na afloop van uw project.

Wijziging Algemene subsidiebepalingen ZonMw
De Algemene subsidiebepalingen ZonMw 2013, zijn per 1 april 2022 gewijzigd. De wijzigingen hebben met name betrekking op artikel 25 subsidievaststelling en artikel 26 verantwoording. U kunt de subsidiebepalingen en wijziging nalezen op de ZonMw website.

1. Aandachtspunten programmacommissie

De programmacommissie heeft de volgende aandachtspunten waarop u in het eerste voortgangsverslag moet reageren:

- De commissie ziet graag dat nog een 2e GGZ instelling betrokken wordt bij het onderzoek.
- In verband met het bewaken van de veiligheid voor de patiënt raadt de commissie aan een interim analyse uit te voeren (met duidelijk vooraf gespecialiseerde stop criteria) en een Data Safety Montitoring Board (DSMB) in te stellen.

Algemene aandachtspunten:

- Tips over het optimaliseren van patiënteninclusie na te lezen op de website van het Qualitative Research Integrated within Trials (QuinteT) team van de University of Bristol en deze ter harte te nemen voor uw studie;
- In lijn met de WHO richtlijn voor het openbaar maken van resultaten van klinische trials zet ZonMw in op snelle toegankelijkheid van de onderzoeksresultaten. Dit betekent dat binnen 12 maanden na afronding van het onderzoek de hoofdresultaten publiekelijk toegankelijk dienen te zijn via het klinisch trial register.
- Om een helder inzicht te geven in de instroom en doorstroom vragen we u gebruik te maken van de CONSORT-richtlijnen. Bij het voortgangsverslag vragen we u een CONSORT Flow-diagram toe te voegen; deze krijgt u bij het opvragen van het voortgangsverslag door ZonMw toegestuurd per email.
- De leidraad “Nieuwe interventies in de klinische praktijk” te volgen, indien van toepassing voor uw project. Deze vindt u onder andere via de website van het Kennisinstituut van Medisch Specialisten.

2. Start en verantwoording van uw project

2.1 Start project

Het project moet uiterlijk 31 december 2022 starten. Alleen in bijzondere gevallen kan hiervan worden afgeweken. Neem hiervoor voor 31 december 2022 contact op met uw contactpersoon bij ZonMw.

2.2 Betaling van de subsidie

De subsidie zullen we via voorschotbedragen aan u overmaken. Als er aan alle door ZonMw gestelde voorwaarden is voldaan, ontvangt u rond de startdatum van het project het eerste voorschot.

De volgende betalingen zijn afhankelijk van de ontvangst, goedkeuring van het voortgangsverslag en de eindverantwoording van uw project.

2.3 Voortgangsverslag
ZonMw wil graag op de hoogte blijven van de voortgang van uw project. Van het programmasecretariaat ontvangt u rond 18 maanden na startdatum het verzoek een voortgangsrapportage in te dienen.

2.4 Tussentijdse wijzigingen
Als er tussentijds iets in uw plan of uw organisatie verandert, moet u dit bij ons melden. Verandering in de opzet, planning, begroting of organisatie kunnen gevolgen hebben voor uw subsidie. Pas na goedkeuring door ZonMw zijn eventuele wijzigingen toegestaan.

2.5 Eindverantwoording
Binnen 13 weken na afloop van uw project dient u een eindverantwoording in.

- Bij projecten met een subsidie onder de € 25.000 bestaat de eindverantwoording uit een inhoudelijk eindverslag.
- Bij projecten met een subsidie tussen de € 25.000 en € 125.000 bestaat de eindverantwoording uit:
    - een inhoudelijk eindverslag;
    - een financiële verantwoording;
    - aanvraag tot subsidievaststelling.
- Bij projecten met een subsidie van € 125.000 of meer bestaat de eindverantwoording uit een:
    - inhoudelijk eindverslag;
    - een financiële verantwoording;
    - een controleverklaring van een accountant;
    - aanvraag tot subsidievaststelling.

  Neem het bedrag voor accountantskosten op in uw eindverantwoording met een maximum van € 3.500.

Voor het inhoudelijk eindverslag moet u gebruik maken van het format dat u, als het zover is, krijgt toegestuurd door het programmateam. De financiële verantwoording moet een specificatie van de verschillende posten bevatten. Voor de aanvraag tot subsidievaststelling moet u gebruik maken van het format op de ZonMw website. Na ontvangst en goedkeuring van de eindverantwoording door ZonMw vindt de definitieve subsidievaststelling en afrekening plaats op basis van werkelijk gemaakte kosten.

2.6 Registratie NTR
Bij patiëntgebonden onderzoek vraagt ZonMw om de gegevens van de studie aan te melden bij het Nederlands Trial Register (NTR). Zie informatie op www.trialregister.nl.

3. Samenwerking en voorkomen van onrechtmatige staatssteun

3.1 Samenwerking en bijdragen van derden
Op de ZonMw-webpagina Subsidies en Samenwerking/bijdragen van derden vindt u meer informatie, voorbeelden van en de voorwaarden voor het opstellen van een samenwerkings- en/of sponsorovereenkomst. Wij verzoeken u deze voorbeeldovereenkomsten en informatie te gebruiken bij het opstellen van de betreffende overeenkomst voor zover van toepassing. Maak hierbij duidelijk wat de wijzigingen ten opzichte van de voorbeeldovereenkomst zijn. De overeenkomst wordt beoordeeld op conformiteit aan het toepasselijke Europese staatssteunrecht, de Algemene subsidiebepalingen ZonMw en de voorwaarden van de subsidieoproep. Acceptatie door ZonMw van de concept samenwerkings- en/of sponsorovereenkomst ontslaat de subsidieontvanger

niet van zijn eigen verantwoordelijkheid met betrekking tot het voorkomen van schending van het Europese staatssteunrecht. Na acceptatie van de concept samenwerkings- en/of sponsorovereenkomst door ZonMw (voor zover die is opgevraagd) en voor de start van het project stuurt u de volledig ondertekende overeenkomst naar ZonMw. De startdatum van het project mag niet eerder zijn dan de datum bij de laatste handtekening op de overeenkomst.

3.2 Voorkomen van onrechtmatige staatssteun

ZonMw verleent deze subsidie met toepassing van het Vrijstellingsbesluit voor diensten van algemeen economisch belang (hierna: het DAEB Vrijstellingsbesluit)[8].

ZonMw heeft de uit te voeren projectactiviteiten als een Dienst van Algemeen Economisch Belang (hierna: "DAEB") gekwalificeerd. Voor de uitvoering van deze projectactiviteiten belast ZonMw het consortium/projectteam/de samenwerkende partijen door middel van een aanwijzingsbesluit met een DAEB (zie bijlage 2 voor het DAEB aanwijzingsbesluit).
Naast de Algemene subsidiebepalingen ZonMw gelden ook de voorwaarden en verplichtingen uit het DAEB Vrijstellingsbesluit zoals opgenomen in het hierboven genoemde aanwijzingsbesluit en andere toepasselijke Europese wet- en regelgeving.

3.3 Opdrachtverlening

Is er sprake van inhuur van of opdrachtverlening aan derden? Dan moeten deze afspraken voldoen aan de voorwaarden voor inhuur of opdrachtverlening zoals vermeld op de ZonMw-webpagina Subsidies en Samenwerking/bijdragen van derden.

4. Publicatie projectresultaten

U moet ZonMw tijdens en tot 4 jaar na afronding van uw project via Mijn ZonMw informeren over producten, publicaties en andere resultaten van uw project.

4.1 Open Access publiceren

Alle publicaties die voortkomen uit wetenschappelijk onderzoek dat geheel of gedeeltelijk door ZonMw gesubsidieerd is, moeten direct (zonder embargo) Open Access beschikbaar gesteld worden, overeenkomstig met het ZonMw Open Access beleid. ZonMw accepteert verschillende Open Access routes. Naast artikelen, moedigt ZonMw ook aan om andere type wetenschappelijke publicaties Open Access beschikbaar te stellen. Voor meer informatie over het ZonMw Open Access beleid en het beleidsdocument zie: www.zonmw.nl/nl/over-zonmw/open-science-fair-data/open-access/.

Als u Open Access publicatiekosten heeft opgenomen in de projectbegroting moet u na publicatie de naam van het journal en een bewijs van betaling (Article Processing Charges) mailen naar doelmatigheidsonderzoek@zonmw.nl en openscience@zonmw.nl.

4.2 Creative Commons licentie

Publicaties moeten gepubliceerd worden onder toepassing van de *Creative Commons* Naamsvermelding (CC BY 4.0 licentie). Dit betekent dat op de auteursversie van deze publicaties (tenzij in de subsidieoproep anders is bepaald) een onherroepelijke, niet-exclusieve CC BY licentie van toepassing is. U moet van het bestaan van deze licentie melding maken aan elke partij

[8]Besluit van de Commissie van 20 december 2011 betreffende de toepassing van artikel 106, lid 2, van het Verdrag betreffende de werking van de Europese Unie op staatssteun in de vorm van compensatie voor de openbare dienst, verleend aan bepaalde met het beheer van diensten van algemeen economisch belang belaste ondernemingen (C(2011) 9380).

met wie u in overleg treedt omtrent publicatie van (een deel van de) projectresultaten. ZonMw is te allen tijde gerechtigd aan derden melding te maken van deze licentie.

4.3 Europe Pubmed Central (EPMC)
Voor alle subsidierondes gepubliceerd vanaf 01-01-2021 vereist ZonMw dat projectleiders tenminste een kopie (*Version of Record* of het *Author Accepted Manuscript*) van artikelen voortkomend uit hun ZonMw-project deponeren in EPMC (zelfdeponering of via uitgever).
In verband met de koppeling die in EPMC wordt gemaakt tussen publicaties en subsidie en projectinformatie van door ZonMw gefinancierde projecten, worden met enige regelmaat algemene projectgegevens en enkele persoonsgegevens gelinkt aan het project (naam projectleider, e-mailadres (*enkel voor het maken van de koppeling, dit wordt niet verder gedeeld of gebruikt*), organisatie en afdeling) gedeeld met EMBL-EBI, de beheerder van EPMC. ZonMw heeft hiervoor een officiële *data sharing agreement* afgesloten. Mocht u hier bezwaar tegen hebben, dan vernemen wij dit graag uiterlijk *twee weken* na ontvangst van deze toekenningsbrief. Indien wij niets van u vernemen, gaan wij ervan uit dat u akkoord bent met deze verwerking van uw persoonsgegevens.

4.4 Licentie of overdracht van projectresultaten
Bij de uitvoering van uw project en het gebruik van (toekomstige) resultaten moet u de principes van Maatschappelijk Verantwoord Licentiëren toepassen.

5. Integriteit
Het project moet u op een goede en integere manier uitvoeren. Hiervoor gelden onder andere de volgende principes: eerlijkheid, zorgvuldigheid, transparantie, onafhankelijkheid en verantwoordelijkheid. Deze principes zijn vastgelegd in de Nederlandse gedragscode wetenschappelijke integriteit. Als er bij uw project een (mogelijke) integriteitsschending plaatsvindt, stelt u ZonMw hier zonder uitstel van op de hoogte. Hierbij moet u alle relevant documenten over de integriteitsschending aan ZonMw overhandigen.

6. Communicatie over uw project
ZonMw hecht veel waarde aan het delen van de voortgang, opbrengsten en resultaten van gehonoreerde projecten. Als uw project in de media verschijnt, geef dit dan aan ons door. Wij plaatsen dit graag op uw ZonMw-projectpagina en kunnen het ook verspreiden via onze communicatiekanalen.
Geef daarnaast in alle communicatie-uitingen over uw project aan dat uw project mogelijk wordt gemaakt door ZonMw. Meer informatie hierover en het logo van ZonMw vindt u op www.zonmw.nl/huisstijl.

Bijlage 2 - DAEB aanwijzingsbesluit

Gelet op

- artikel 14 en artikel 106 lid 2 van het Verdrag betreffende de Werking van de Europese Unie (hierna: "VWEU");
- het besluit van de Commissie, nr.2012/21/EU van 20 december 2011 betreffende de toepassing van artikel 106 lid 2 van het VWEU op staatssteun in de vorm van compensatie voor de openbare dienst, verleend aan bepaalde met het beheer van diensten van algemeen economisch belang belaste ondernemingen, kennisgegeven onder nummer C(2011)9380 (hierna: "DAEB Vrijstellingsbesluit");
- de Wet op de organisatie ZorgOnderzoek Nederland van 14 februari 1998 (Stb. 1998, 124) waarin is vastgelegd dat ZorgOnderzoek Nederland tot taak heeft het doen uitvoeren en het subsidiëren of het verlenen van opdrachten met betrekking tot projecten, experimenten, onderzoek en ontwikkeling op het terrein van gezondheid, preventie en zorg;

Overwegende dat

- de binnen het project uit te voeren activiteiten door ZonMw worden aangemerkt als een Dienst van Algemeen Economisch Belang (hierna: “DAEB”) in de zin van artikel 106 lid 2 van het VWEU, zoals opgenomen en onderbouwd in de subsidieoproep ‘Open ronde 2023, onderzoek naar de effectiviteit en kosten van interventies’
- het aanwijzen van de activiteiten als een DAEB binnen het project *Higher, faster, better: a study on (cost-) effectiveness of neuromodulation in depression. Is an accelerated intermittent Theta Burst Stimulation protocol compared to standard 10 Hz repetitive Transcranial Magnetic Stimulation, more (cost-) effective in patients with treatment-resistant depression?*, onderdeel is van het streven van de overheid om het geconstateerde marktfalen te adresseren;
- om gebruik te kunnen maken van de bijzondere positie die een DAEB inneemt binnen de Europese mededingingsregelgeving, een onderneming daarvoor specifiek met het beheer van een bepaalde DAEB dient te worden belast;

Besluit ZonMw om:

1. **Consortium en activiteiten**

Het consortium in de projectaanvraag, waarbij de hoofdaanvrager is gevestigd aan De Boelelaan 1117 1081 HV Amsterdam, met het KvK nummer 64156338 te belasten met het verrichten van een DAEB bestaande uit de activiteiten zoals beschreven in de projectaanvraag *“Higher, faster, better: a study on (cost-) effectiveness of neuromodulation in depression. Is an accelerated intermittent Theta Burst Stimulation protocol compared to standard 10 Hz repetitive Transcranial Magnetic Stimulation, more (cost-) effective in patients with treatment-resistant depression?”*

De aanwijzing van de DAEB vangt aan op de startdatum van het project en duurt de gehele looptijd van het project (met een maximum van 10 jaar). Het project dient uiterlijk 31 december 2022 van start te gaan.

Nieuwe partij(en) toevoegen aan het consortium
Indien op een later moment een nieuwe partij zal worden toegevoegd aan het consortium, dient u dit schriftelijk te melden bij ZonMw. De nieuwe partij zal pas onderdeel vormen van het consortium indien ZonMw goedkeuring heeft verleend. Voor alle partijen – dus ook de partijen die op een later moment worden toegevoegd aan het consortium – geldt dat ze afzonderlijk aan alle voorwaarden van het DAEB Vrijstellingsbesluit en de hierin beschreven verplichtingen en voorwaarden dienen te voldoen.

2. **Duur van de DAEB**
De aanwijzing van de DAEB vangt aan op de startdatum van het project en duurt de gehele looptijd van het project (met een maximum van 10 jaar). Het project dient uiterlijk 31 december 2022 van start te gaan.

3. **Toekenning van uitsluitende of bijzondere rechten**
- Niet van toepassing

4. **Overige verplichtingen & voorwaarden**
De DAEB zal bestaan uit het uitvoeren van de in de projectaanvraag beschreven activiteiten. Het subsidiebedrag mag alleen ingezet worden voor de activiteiten die onder de DAEB vallen.

Partijen die binnen deze subsidieronde subsidie ontvangen zijn op grond van artikel 5 lid 2 van het DAEB Vrijstellingsbesluit verplicht om in hun boekhouding de kosten en de baten die samenhangen met de DAEB activiteiten gescheiden op te nemen van kosten en baten van activiteiten die niet onder de DAEB vallen.

De financiering van het project zal de maximale duur van het project niet overschrijden. De maximale duur van een project zal in lijn met het DAEB Vrijstellingsbesluit in ieder geval niet meer dan 10 jaar bedragen.

Het aangevraagde subsidiebedrag mag niet meer bedragen dan de nettokosten van de voorziene projectactiviteiten. De parameters voor de berekening van de compensatie voor elk project zijn opgenomen in de ingediende begroting. De berekeningswijzen opgenomen in de ingediende begroting zijn in overeenstemming met artikel 4 van het DAEB Vrijstellingsbesluit. Gebleken overcompensatie vordert ZonMw op grond van artikel 6 lid 2 van het DAEB Vrijstellingsbesluit terug.

Is de looptijd van het project langer dan 3 jaar dan kan ZonMw een tussentijdse controle uitvoeren of er sprake is van overcompensatie. Indien bij controle blijkt dat de door aanvragers ontvangen subsidie hoger is dan de nettokosten, zal ZonMw het surplus terugvorderen. Deze controle behelst de hele looptijd van het project of vanaf het moment van de laatste tussencontrole.

Indien blijkt dat de projectactiviteiten niet, of niet geheel zijn verricht, dan wel niet, of niet geheel aan de subsidie verbonden verplichtingen is voldaan, kan ZonMw de subsidie op een lager bedrag vaststellen en uitbetaalde voorschotten(deels) terugvorderen.
