# Supplementary material for: Comparison of 5-Day Multidaily Neuronavigated Theta-Burst Sessions With 6-Week Standard Repetitive Transcranial Magnetic Stimulation (the Dutch Depression Outcome Trial): Protocol for a Randomized Controlled Trial
Source: JMIR Res Protoc. 2025 Aug 21;14:e70121. doi: 10.2196/70121 (PMC12411794; doi:10.2196/70121)
Supplement: Multimedia Appendix 2 [file resprot_v14i1e70121_app2.pdf]

Beoordelingsformulier referent - DoelmatigheidsOnderzoek Open ronde 2023 UA 2022

Referentbeoordeling

1. Introduction

1.1 Details	
**Grant programme:**	DoelmatigheidsOnderzoek
**Project number:**	10390012110079
**Applicant:**	Annemiek Dols
**Project title:**	Higher, faster, better: a study on (cost-) effectiveness of neuromodulation in depression. Is an accelerated intermittent Theta Burst Stimulation protocol compared to standard 10 Hz repetitive Transcranial Magnetic Stimulation, more (cost-) effective in patients with treatment-resistant depression?
**Code report:**	3635953

1.2 Conflict of interest and Confidentiality

Should you agree to assess this application, the Netherlands Organization for Health Research and Development (ZonMw) will assume that you can be regarded as an independent expert in the relevant field without personal interests as referred to in the Code for dealing with Personal Interests . If you have any doubts in this respect, please contact the programme team.

The procedures governing assessments require strict confidentiality. This means only that reviewers’ assessments are always anonymized, but also that reviewers must handle all proposals strictly confidentially.

☑ I can be regarded as an independent expert, without any conflict of interest, in the relevant field, as referred to in the Code for dealing with personal interests https://www.zonmw.nl/en/about-zonmw/integrity-and-conflicts-of-interest/

☑ I declare that I will keep the content of the proposal and all additional information in this review process strictly confidential.

1.3 Instruction

For each section or sub-section, please describe both positive and negative aspects with regard to the listed criteria. For the overall quality assessment, please reread your comments in the boxes, give a score and summarise the main strengths and weaknesses of the proposal on which your score was based.

The following guidelines may help you when assigning scores while reviewing the grant application(s):

- **Excellent. The proposal successfully addresses all aspects of the criterion in question.**
- **Good. The proposal addresses the criterion in question well but certain improvements are necessary.**
- **Sufficient. The proposal generally addresses the criterion, but there are significant weaknesses that need corrections.**
- **Poor. The proposal shows weaknesses in relation to the criterion in question.**
- **Insufficient. The proposal shows serious weaknesses in relation to the criterion in question.**

Your review is an important contribution to the assessment of the application and will have a significant influence on the final funding decision. We would therefore greatly appreciate it if you could elaborate your scoring with in-depth comments and/or suggestions.

Please refer to the assessment criteria in such a way that your comments are consistent and informative for the applicant and the assessment committee.

The committee will make the final quality assessment based on the grant application, the reviewers' opinions and the applicant's response. The committee also assesses the relevance and priority of applications in terms of the programme specific criteria.

Your assessment will be sent to the applicant in anonymous form. They will then have an opportunity to respond. The committee will also receive an anonymous version of your assessment. We would, therefore, urge you to avoid making any references to yourself in your reviewer's report.

2. Reviewform

2.1 Objective and problem definition

Please assess the quality of the objective(s) and problem definition.

Consider the following factors:

- **How clear the objective is**
- **How clear and verifiable the problem definition is and whether it is consistent with the objective**
- **There is a clear description of the expected outcomes and intended results**
- **The value added to existing knowledge or practice**
- **The theoretical or empirical evidence presented in support of the problem definition**

**Score:**	Excellent

Please indicate positive and negative aspects and address the major and minor negative aspects separately.

Positive aspects:

The Objective is clear and simple, this grant aims at investigating the possible superiority of a new rTMS protocol that could 1) improve remission rate 2) reduce cost and time. This investigation is needed as the only existing literature does not directly compare the new protocol to the classic rTMS protocol and relies on very small cohort of patients. A clear superiority of the SNT protocol demonstrated by this study would likely promote the use of this protocol and benefit thousand of patients.

Negative aspects:

With such a cohort of patients, it is disappointing to see that no other investigations appear to be planned such as an evaluation of possible changes pre-post treatment (behavioral/neuroimaging changes). Given the reliance of the SNT on functional connectivity mapping, one could expect a post treatment resting state to be acquired for example. Of course this is difficult given the multicenter nature of this study.

2.2 Adequacy of the clinical study design

Please assess the adequacy of the clinical study design.

In the call for proposals extra attention is given to the adequacy of the chosen design in relation to the research question. Researchers should be able to explain how the proposed research method is appropriate for the question posed, demonstrate that the design will answer the research question, and why it is the most adequate approach. Researchers should specify the setting in which the research is conducted. For instance, is it feasible to randomize the patients if the research comparators are different in kind (e.g. surgery versus behavioral therapy)? Is it possible to have a control group using standard care?

Consider the following factors:

- **Is the chosen design well motivated in the grant proposal?**
- **Is the chosen design appropriate for the question posed and will this design answer the research question?**

**Score:**	Goed

Please indicate positive and negative aspects and address the major and minor negative aspects separately.

Positive aspects:

The chosen design is adapted to the objective of the study and the treatment(s) proposed.

Negative aspects:

There are a few negative aspects to such design but they are almost impossible to prevent in TMS studies: 1) the lack of (adequate) blinding : in this design blinding of the operator is extremely difficult, if not impossible to achieve. 2) the lack of placebo comparison: it is always an added benefit to have a placebo branch, but this would complicate the studies and increase the cost significantly.
More specifically to this proposal, patients not responding might want to receive the other protocol, it is not clear how this will be implemented and what data will be collected (if any).

2.3 Methods of the clinical study

Please assess the methods of the clinical study.

Consider the following factors:

- Eligibility criteria for participants
- Adequacy of the intervention for each group and description of the intervention with sufficient details
- Adequacy of outcomes defined as primary (patient oriented) and secondary outcome measures
- Adequacy of the sample size calculation
- Adequacy of statistical methods used to compare groups for primary and secondary outcomes
- Relevant differences within target groups (gender, ethnicity, age and/or other relevant characteristics) and if applicable methods that will be used for additional (subgroup) analyses

**Score:**	Voldoende

Please indicate positive and negative aspects and address the major and minor negative aspects separately.

Positive aspects:

The eligibility criteria for participants are standards and adapted to the objective of the study. The 2 interventions proposed are well known and described.
The outcomes measure are also standard and adapted to the aim of the study.
The sample size seem adequate and the statistical methods well thought.

Negative aspects:

There is no mention of response rate, only remission. Another important missing outcome measure could be suicidal ideation.
The Last Observation Carry Forward approach is outdated and relies on unrealistic assumptions, especially in the field of psychiatry.

2.4 Cost-effectiveness analysis

Please assess the cost-effectiveness analysis.

The purpose of the cost-effectiveness analysis (CEA) is to assess the proposed gain in health care efficiency of the (new) intervention(s) compared to the usual care provided in the Netherlands.

Consider the following factors:

- Whether all elements of PICOT (patients, intervention, comparator(s), outcomes, time horizon) are clearly stated in the cost-effectiveness analysis
- Whether the CEA is well designed
- Whether the methods of data collection are appropriate
- Whether the results are generalizable to other relevant settings
- For Dutch referees: is it consistent with the latest Dutch guidelines for health economic evaluations?

**Score:**	Voldoende

Please indicate positive and negative aspects and address the major and minor negative aspects separately.

Positive aspects:

The CEA and data collection are well thought.

Negative aspects:

The whole procedure should be better explained. It is my impression that the SNT protocol requires an fMRI session to be acquired for each participant. This is not included in the cost of the SNT as I believe it is not considered for reimbursement yet. Is my understanding correct ?
How long is the required fMRI session, how much does it cost? Ultimately the patients' perspective has to be considered too when judging for the cost/effectiveness of a treatment. Not only the Healthcare / societal perspective.

2.5 Budget impact analysis

Please assess the budget impact analysis.

The purpose of the budget impact analysis (BIA) is to assess the financial consequences of dissemination of the (new) intervention(s). Information obtained from the BIA can be used for policy decisions on a national, regional and/or local level. Various perspectives can be taken into account; the government and insurance perspectives must always be considered.

Consider the following factors:

- Clarity
- Well-designed in terms of perspectives, scenarios, population, time horizon, etc.?

- Are all relevant cost assessments included?
- Appropriate data collection?
- For Dutch referees: is it consistent with the latest Dutch guidelines for health economic evaluations?

**Score:**	Excellent

Please indicate positive and negative aspects and address the major and minor negative aspects separately.

Positive aspects:

The BIA appears to be complete and well designed, every relevant scenarios will be investigated following accepted methods.

Negative aspects:

NA

2.6 Feasibility of the project

Please assess the feasibility of the study.

It is known that many projects in the Health Care Efficiency Research Program have a considerable delay and not always are the objectives achieved. In the grant proposal it should be motivated that the project is feasible within the proposed timeframe and budget.

Consider the following factors:

- Prospects of achieving the objective or objectives using this strategy
- Research protocol
- Realistic phasing and timetable
- Availability of facilities/staff
- Realistic number of patients/institutes/organisations
- Is the number of potential respondents to be approached in participating centres sufficient?
- Has the expected response been properly estimated? What is the non-response?
- Recruitment/participation of patients and if applicable: is randomisation feasible?

**Score:**	Excellent

Please indicate positive and negative aspects and address the major and minor negative aspects separately.

Positive aspects:

The study time frame is extremely realistic, especially spread over multiple centers and should not negatively impact the normal activity of the centers.

Negative aspects:

I believe response should also be assessed as response and not only remission, ie a significant decrease in symptoms has a real impact on patients life.
The timeline of the 2nd protocol being provided for the patients not responding is not very clear.

2.7 Systematic review

Please assess the systematic review.

See the appendix Systematic review.

Consider the following factors:

- Selection of search terms
- Are all relevant databases included?
- Selection of papers
- Are any references relevant to this specific proposal missing?
- Are the conclusions of the systematic review justified?

**Score:**	Excellent

Please indicate positive and negative aspects and address the major and minor negative aspects separately.
**Positive aspects:**
The systematic review has been conducted under reasonable search terms, using the relevant databases and by selecting important landmarks papers. The conclusion are justified.
**Negative aspects:**
NA

2.8 Project group

Please assess the project group.

Consider the following factors:

- Relevant (multidisciplinary) expertise
- Familiarity with the discipline/research area
- Prior activities and products
- Ability of the group to successfully complete the proposed project
- Division of roles and tasks at implementation level

Score:	Excellent
**Please indicate positive and negative aspects and address the major and minor negative aspects separately.**	
**Positive aspects:**	
The project group includes neuroscientists, neuropsychiatrists, neuroanatomists, economists and statisticians. The required expertise is definitely present, in addition the group is appears to be expert in the field of TMS and depression with more than 750 publications,	
**Negative aspects:**	
The group feels dominated by clinicians which could explain the lack of interest for secondary measures.	

2.9 Overall quality assessment

Please assess the entire application with regard to the quality of the proposal based on the elements above.

Score:	Goed
**Please summarise your overall findings regarding the main strengths and weaknesses of the proposal. Please make sure your score is in line with your arguments.**	
**Strengths:**	
The principal quality of this proposal is its importance and simplicity. If SNT proves to be superior and more cost efficient than 10Hz rTMS, then this study could not only save important amount of money but most importantly help thousands of patients.<br>Another strength is that the charge of work is well split between closely located centers which will help the study run smoothly and improve communication.	
**Weaknesses:**	
While 10Hz rTMS remains the dominant treatment, iTBS is increasingly proposed across the world. It feels like a missed opportunity to compare the 3 protocols (SNT, iTBS, 10hz) . Of course this would complicate the design and increase the needed number of patients. In the same line, it is disappointing than no placebo control group is proposed but I understand that there are no perfect study and concessions have to be made.	

2.10 Budget

Please give your opinion on the budget based on the data supplied in the grant application. Important for the judgement of the budget is that usual care covered by Dutch health insurance is not chargeable to the grant. There is a comprehensive overview of the requested budget (in Dutch).

Please note that your assessment of these criteria should not be taken into account in your quality score above.

Consider the following factors:

- The requested budget is realistic for this project proposal.

- **The requested budget contains an extensive overview of activities that need to be funded.**
- **The requested budget (and co-financing) is clearly explained.**
- **The requested budget is appropriate given the nature of the product and the need for highly qualified staff.**
- **Cost-benefit ratio (the balance between effort, input, use of resources and the likely outcomes and yield)**

**Score:**	Realistisch
**If possible, please indicate positive and negative aspects and address the major and minor negative aspects separately.**	
**Positive aspects:**	
The numbers seem realistic but it is difficult for me to judge given that I am located in a different country.	
**Negative aspects:**	
The numbers seem realistic but it is difficult for me to judge given that I am located in a different country.<br>No TMS coil budget ? What is a TMS coil fails and need replacement ?	

Beoordelingsformulier referent - DoelmatigheidsOnderzoek Open ronde 2023 UA 2022

Referentbeoordeling

1. Introduction

1.1 Details	
**Grant programme:**	DoelmatigheidsOnderzoek
**Project number:**	10390012110079
**Applicant:**	Annemiek Dols
**Project title:**	Higher, faster, better: a study on (cost-) effectiveness of neuromodulation in depression. Is an accelerated intermittent Theta Burst Stimulation protocol compared to standard 10 Hz repetitive Transcranial Magnetic Stimulation, more (cost-) effective in patients with treatment-resistant depression?
**Code report:**	3708901

1.2 Conflict of interest and Confidentiality

Should you agree to assess this application, the Netherlands Organization for Health Research and Development (ZonMw) will assume that you can be regarded as an independent expert in the relevant field without personal interests as referred to in the Code for dealing with Personal Interests . If you have any doubts in this respect, please contact the programme team.

The procedures governing assessments require strict confidentiality. This means only that reviewers' assessments are always anonymized, but also that reviewers must handle all proposals strictly confidentially.

☑ I can be regarded as an independent expert, without any conflict of interest, in the relevant field, as referred to in the Code for dealing with personal interests https://www.zonmw.nl/en/about-zonmw/integrity-and-conflicts-of-interest/

☑ I declare that I will keep the content of the proposal and all additional information in this review process strictly confidential.

1.3 Instruction

For each section or sub-section, please describe both positive and negative aspects with regard to the listed criteria. For the overall quality assessment, please reread your comments in the boxes, give a score and summarise the main strengths and weaknesses of the proposal on which your score was based.

The following guidelines may help you when assigning scores while reviewing the grant application(s):

- **Excellent. The proposal successfully addresses all aspects of the criterion in question.**
- **Good. The proposal addresses the criterion in question well but certain improvements are necessary.**
- **Sufficient. The proposal generally addresses the criterion, but there are significant weaknesses that need corrections.**
- **Poor. The proposal shows weaknesses in relation to the criterion in question.**
- **Insufficient. The proposal shows serious weaknesses in relation to the criterion in question.**

Your review is an important contribution to the assessment of the application and will have a significant influence on the final funding decision. We would therefore greatly appreciate it if you could elaborate your scoring with in-depth comments and/or suggestions.

Please refer to the assessment criteria in such a way that your comments are consistent and informative for the applicant and the assessment committee.

The committee will make the final quality assessment based on the grant application, the reviewers' opinions and the applicant's response. The committee also assesses the relevance and priority of applications in terms of the programme specific criteria.

Your assessment will be sent to the applicant in anonymous form. They will then have an opportunity to respond. The committee will also receive an anonymous version of your assessment. We would, therefore, urge you to avoid making any references to yourself in your reviewer's report.

2. Reviewform

2.1 Objective and problem definition

Please assess the quality of the objective(s) and problem definition.

Consider the following factors:

- **How clear the objective is**
- **How clear and verifiable the problem definition is and whether it is consistent with the objective**
- **There is a clear description of the expected outcomes and intended results**
- **The value added to existing knowledge or practice**
- **The theoretical or empirical evidence presented in support of the problem definition**

**Score:**	Excellent

Please indicate positive and negative aspects and address the major and minor negative aspects separately.

Positive aspects:

The objective of this study is to compare standard repetitive TMS versus a newer form of accelerated TMS, called Stanford Neuromodulation Therapy (SNT). In brief, standard TMS uses anatomical landmarks to target the dorsolateral prefrontal cortex, with treatments delivered on a daily basis over 30 sessions (typically six weeks). The major differences in SNT are 1) neuronavigated coil placement, 2) delivery of total treatment (90,000 pulses) over five days, and 3) different device parameters, using theta burst TMS. SNT has several small, unblinded studiess supporting its use, and a single very small (N=32) randomized controlled trial where active SNT was superior to sham SNT. However, the available evidence is insufficient to make any meaningful conclusions about the real-world clinical effectiveness or potential cost effectiveness. This current proposal would address this need. If SNT is clearly superior in acute and longer-term outcomes, it will lead to a markedly different approach to TMS use.

Negative aspects:

None; the question under study is important to the field, and the proposed study is well-poised to answer it (assuming other items, identified below, are resolved).
Under Motive - if the motivation is to find a replacement for ECT, the study should compare SNT versus ECT and not standard rTMS.

2.2 Adequacy of the clinical study design

Please assess the adequacy of the clinical study design.

In the call for proposals extra attention is given to the adequacy of the chosen design in relation to the research question. Researchers should be able to explain how the proposed research method is appropriate for the question posed, demonstrate that the design will answer the research question, and why it is the most adequate approach. Researchers should specify the setting in which the research is conducted. For instance, is it feasible to randomize the patients if the research comparators are different in kind (e.g. surgery versus behavioral therapy)? Is it possible to have a control group using standard care?

Consider the following factors:

- **Is the chosen design well motivated in the grant proposal?**
- **Is the chosen design appropriate for the question posed and will this design answer the research question?**

**Score:**	Goed

Please indicate positive and negative aspects and address the major and minor negative aspects separately.

Positive aspects:

The chosen design is well-motivated and appropriate.
Randomization is appropriate; patients' preferences are also recorded.
Identification of short, medium, and longer-term effectiveness is critically important - this is measured by relapse at 4, 10- and 25-weeks post treatment.

Negative aspects:

Clinical outcomes using last-observation carried forward is not appropriate; mixed models are most appropriate as they are more robust and retain the temporal features of clinical changes over time.
It is not possible to use a sham given the vast differences between the two protocols. Blinded raters are used, but there was little detail about how the raters would be blinded. A best blind would be blinded to group assignment and treatment interval.
SNT requires neuronavigated stimulation - navigated to a point of maximal resting state functional connectivity anticorrelation to the subgenual ACC- how will multisite MRI efforts be standardized with adequate quality control?
SNT used a depth-adjusted motor threshold; this is not mentioned in the application
The SNT approach used a proprietary software to identify the optimal stimulation site location - how is the to be addressed?

2.3 Methods of the clinical study

Please assess the methods of the clinical study.

Consider the following factors:

- **Eligibility criteria for participants**
- **Adequacy of the intervention for each group and description of the intervention with sufficient details**
- **Adequacy of outcomes defined as primary (patient oriented) and secondary outcome measures**
- **Adequacy of the sample size calculation**
- **Adequacy of statistical methods used to compare groups for primary and secondary outcomes**
- **Relevant differences within target groups (gender, ethnicity, age and/or other relevant characteristics) and if applicable methods that will be used for additional (subgroup) analyses**

**Score:**	Voldoende

Please indicate positive and negative aspects and address the major and minor negative aspects separately.

Positive aspects:

Eligibility criteria generally are appropriate, and the adequacy of the interventions are appropriate
Outcomes are appropriate and patient oriented.

Negative aspects:

Sample size and power calculations are likely too optimistic. Attrition of <10% (cited as 7% in the application) is too low and should be assumed to be 15%.
Statistical methods are not appropriate - see comment above - last observation carried forward is not appropriate.
How is missing data handled?
Randomization should be stratified, at least by sex. The team should consider additionally stratifying patients by baseline/pretreatment symptom severity.
Consider use of a modified ITT design, to include participants randomized and who received at least 1 TMS session.
It was unclear why patients with first-degree relatives with epilepsy would be excluded; that is not common.
Medication stability for 4 weeks is likely to be insufficient; antidepressant medications often take longer to be effective and as such six weeks should be the minimum.
MRI scans to rule out epilepsy is not appropriate - identifying epilepsy is based on a clinical interview, followed by EEG and MRI.
Clinical relapse needs to be clearly defined.
There appears to be an analysis investigating the distance between "optimal" site from RSFC and Beam F3 - so are all patients going to get an MRI? This was not clear.

2.4 Cost-effectiveness analysis

Please assess the cost-effectiveness analysis.

The purpose of the cost-effectiveness analysis (CEA) is to assess the proposed gain in health care efficiency of the (new) intervention(s) compared to the usual care provided in the Netherlands.

Consider the following factors:

- **Whether all elements of PICOT (patients, intervention, comparator(s), outcomes, time horizon) are clearly stated in the cost-effectiveness analysis**
- **Whether the CEA is well designed**
- **Whether the methods of data collection are appropriate**
- **Whether the results are generalizable to other relevant settings**
- **For Dutch referees: is it consistent with the latest Dutch guidelines for health economic evaluations?**

**Score:**	Onvoldoende

Please indicate positive and negative aspects and address the major and minor negative aspects separately.

Positive aspects:

This appears to be appropriate, but as an outsider to Netherlands I cannot adequately judge.

Negative aspects:

No judgement - see above
Note that "MRI is performed before ECT" is included and likely an error from a prior application.

2.5 Budget impact analysis

Please assess the budget impact analysis.

The purpose of the budget impact analysis (BIA) is to assess the financial consequences of dissemination of the (new) intervention(s). Information obtained from the BIA can be used for policy decisions on a national, regional and/or local level. Various perspectives can be taken into account; the government and insurance perspectives must always be considered.

Consider the following factors:

- **Clarity**
- **Well-designed in terms of perspectives, scenarios, population, time horizon, etc.?**
- **Are all relevant cost assessments included?**
- **Appropriate data collection?**
- **For Dutch referees: is it consistent with the latest Dutch guidelines for health economic evaluations?**

**Score:**	Goed

Please indicate positive and negative aspects and address the major and minor negative aspects separately.

Positive aspects:

This appears appropriate, but I am not familiar with elements of the Dutch system. It is clear, and well designed in terms of the various perspectives needed in the more immediate time horizon. Cost assessments appear appropriate.

Negative aspects:

No judgement.

2.6 Feasibility of the project

Please assess the feasibility of the study.

It is known that many projects in the Health Care Efficiency Research Program have a considerable delay and not always are the objectives achieved. In the grant proposal it should be motivated that the project is feasible within the proposed timeframe and budget.

Consider the following factors:

- **Prospects of achieving the objective or objectives using this strategy**
- **Research protocol**
- **Realistic phasing and timetable**
- **Availability of facilities/staff**
- **Realistic number of patients/institutes/organisations**
- **Is the number of potential respondents to be approached in participating centres sufficient?**
- **Has the expected response been properly estimated? What is the non-response?**
- **Recruitment/participation of patients and if applicable: is randomisation feasible?**

**Score:**	Voldoende

Please indicate positive and negative aspects and address the major and minor negative aspects separately.

Positive aspects:

The proposal is likely feasible; standard TMS is well known. The protocols are well designed and the objectives are achievable.
The timetables appear appropriate (but see below), and the facilities and staff are also appropriate. Patient numbers are adequate as written (although likely larger sample sizes may be needed if attrition is >7%, or differences between the two groups are less than anticipated). Recruitment appears appropriate.

Negative aspects:

Are single sites able to randomized to both standard TMS and SNT at the same time? SNT is very resource intensive.
Use of SNT means that a single TMS device is used for most or all of the day, meaning that patients may not be able to participate in both arms at the same time. This may cause significant delays in participation.
Is a month of start up time sufficient to implement SNT? What measures will be used to ensure fidelity across sites - this will also impact start up time.
SNT includes more than neuronavigated TMS - but also the use of resting state functional connectivity. This requires a research grade MRI scan and appropriate calibration across multiple different sites. This will also impact feasibility.

2.7 Systematic review

Please assess the systematic review.

See the appendix Systematic review.

Consider the following factors:

- **Selection of search terms**
- **Are all relevant databases included?**
- **Selection of papers**
- **Are any references relevant to this specific proposal missing?**
- **Are the conclusions of the systematic review justified?**

**Score:**	Excellent

Please indicate positive and negative aspects and address the major and minor negative aspects separately.

Positive aspects:

The systematic review is appropriate, and the conclusions of the review are justified.

Negative aspects:

none

2.8 Project group

Please assess the project group.

Consider the following factors:

- **Relevant (multidisciplinary) expertise**
- **Familiarity with the discipline/research area**
- **Prior activities and products**
- **Ability of the group to successfully complete the proposed project**
- **Division of roles and tasks at implementation level**

**Score:**	Excellent

Please indicate positive and negative aspects and address the major and minor negative aspects separately.

Positive aspects:

The group has the relevant experitse and familiarity with the area. External consultants are appropriate; prior activities of the team demonstrate a track record of excellence.

Negative aspects:

It was unclear from the application whether these parties have worked together in similar efforts.

2.9 Overall quality assessment

Please assess the entire application with regard to the quality of the proposal based on the elements above.

**Score:**	Goed

Please summarise your overall findings regarding the main strengths and weaknesses of the proposal. Please make sure your score is in line with your arguments.

Strengths:

This is an important study that seeks to compare standard TMS versus a novel accelerated form of TMS. The question is very important, from both an clinical effectiveness and cost-effectiveness perspective. Observations from this study will be important, and include both short, medium and longer-term clinical outcomes. The application comes from a group of experienced researchers, augmented by external consultants.

Weaknesses:
Several elements of the clinical trial design require additional attention, including removal of LOCF methods, better description of rater blinding, and consideration of stratification for factors that are relevant to cost effectiveness and patient outcomes. Anticipated attrition rates are likely too optimistic, which will likely lead to issues of missing data. Whether sites are able to administer both standard TMS and SNT at the same time is unknown, and is an important issue given SNT can be resource intensive in the shorter term. Attention to neuroimaging elements - critical to the targeting of SNT - were insufficiently described and needs to be carefully addressed.

2.10 Budget

Please give your opinion on the budget based on the data supplied in the grant application. Important for the judgement of the budget is that usual care covered by Dutch health insurance is not chargeable to the grant. There is a comprehensive overview of the requested budget (in Dutch).

Please note that your assessment of these criteria should not be taken into account in your quality score above.

Consider the following factors:

- **The requested budget is realistic for this project proposal.**
- **The requested budget contains an extensive overview of activities that need to be funded.**
- **The requested budget (and co-financing) is clearly explained.**
- **The requested budget is appropriate given the nature of the product and the need for highly qualified staff.**
- **Cost-benefit ratio (the balance between effort, input, use of resources and the likely outcomes and yield)**

Score:	Geen mening

If possible, please indicate positive and negative aspects and address the major and minor negative aspects separately.
**Positive aspects:**
n/a
**Negative aspects:**
n/a

Beoordelingsformulier referent - DoelmatigheidsOnderzoek Open ronde 2023 UA 2022

Referentbeoordeling

1. Introduction

1.1 Details	
**Grant programme:**	DoelmatigheidsOnderzoek
**Project number:**	10390012110079
**Applicant:**	Annemiek Dols
**Project title:**	Higher, faster, better: a study on (cost-) effectiveness of neuromodulation in depression. Is an accelerated intermittent Theta Burst Stimulation protocol compared to standard 10 Hz repetitive Transcranial Magnetic Stimulation, more (cost-) effective in patients with treatment-resistant depression?
**Code report:**	3648126

1.2 Conflict of interest and Confidentiality

Should you agree to assess this application, the Netherlands Organization for Health Research and Development (ZonMw) will assume that you can be regarded as an independent expert in the relevant field without personal interests as referred to in the Code for dealing with Personal Interests . If you have any doubts in this respect, please contact the programme team.

The procedures governing assessments require strict confidentiality. This means only that reviewers' assessments are always anonymized, but also that reviewers must handle all proposals strictly confidentially.

☑ I can be regarded as an independent expert, without any conflict of interest, in the relevant field, as referred to in the Code for dealing with personal interests https://www.zonmw.nl/en/about-zonmw/integrity-and-conflicts-of-interest/

☑ I declare that I will keep the content of the proposal and all additional information in this review process strictly confidential.

1.3 Instruction

For each section or sub-section, please describe both positive and negative aspects with regard to the listed criteria. For the overall quality assessment, please reread your comments in the boxes, give a score and summarise the main strengths and weaknesses of the proposal on which your score was based.

The following guidelines may help you when assigning scores while reviewing the grant application(s):

- Excellent. The proposal successfully addresses all aspects of the criterion in question.
- Good. The proposal addresses the criterion in question well but certain improvements are necessary.
- Sufficient. The proposal generally addresses the criterion, but there are significant weaknesses that need corrections.
- Poor. The proposal shows weaknesses in relation to the criterion in question.
- Insufficient. The proposal shows serious weaknesses in relation to the criterion in question.

Your review is an important contribution to the assessment of the application and will have a significant influence on the final funding decision. We would therefore greatly appreciate it if you could elaborate your scoring with in-depth comments and/or suggestions.

Please refer to the assessment criteria in such a way that your comments are consistent and informative for the applicant and the assessment committee.

The committee will make the final quality assessment based on the grant application, the reviewers' opinions and the applicant's response. The committee also assesses the relevance and priority of applications in terms of the programme specific criteria.

Your assessment will be sent to the applicant in anonymous form. They will then have an opportunity to respond. The committee will also receive an anonymous version of your assessment. We would, therefore, urge you to avoid making any references to yourself in your reviewer's report.

2. Reviewform

2.1 Objective and problem definition

Please assess the quality of the objective(s) and problem definition.

Consider the following factors:

- **How clear the objective is**
- **How clear and verifiable the problem definition is and whether it is consistent with the objective**
- **There is a clear description of the expected outcomes and intended results**
- **The value added to existing knowledge or practice**
- **The theoretical or empirical evidence presented in support of the problem definition**

**Score:**	Goed

Please indicate positive and negative aspects and address the major and minor negative aspects separately.

Positive aspects:

The objective is very clear and the application is well written. The expected outcome and intended results are clearly articulated and wellsupproted by the review of the literature.
There is the potential for clear value to be added to existing practice of rTMS in treating depression

Negative aspects:

The limitation of the project is addressed by the applicants: it will not be possible to identify how the SNT protocol delivers better outcomes due to the many modified procedures. This limits further investigation of how TMS protocols might be optimised to deliver particular outcomes.

There is no real theoretical evidence to support the SNT protocol - evidence is only empirical (anecdotal) from the small number of participants involved in the previous clinical trials at Stanford. This is a limitation of TMS field in general and does not detract from the goals of this application

2.2 Adequacy of the clinical study design

Please assess the adequacy of the clinical study design.

In the call for proposals extra attention is given to the adequacy of the chosen design in relation to the research question. Researchers should be able to explain how the proposed research method is appropriate for the question posed, demonstrate that the design will answer the research question, and why it is the most adequate approach. Researchers should specify the setting in which the research is conducted. For instance, is it feasible to randomize the patients if the research comparators are different in kind (e.g. surgery versus behavioral therapy)? Is it possible to have a control group using standard care?

Consider the following factors:

- **Is the chosen design well motivated in the grant proposal?**
- **Is the chosen design appropriate for the question posed and will this design answer the research question?**

**Score:**	Excellent

Please indicate positive and negative aspects and address the major and minor negative aspects separately.

Positive aspects:

The comparison between 10Hz standard treatment and the SNT protocol is well designed. Adjustment of protocols to deliver matching pulse numbers is a strength of the design.
The chosen design is appropriate for the question posed and will answer the research question of which protocol is more efficacious and more cost-effective

Negative aspects:

None that I can see that have not been considered and addressed by the applicants

2.3 Methods of the clinical study

Please assess the methods of the clinical study.

Consider the following factors:

- Eligibility criteria for participants
- Adequacy of the intervention for each group and description of the intervention with sufficient details
- Adequacy of outcomes defined as primary (patient oriented) and secondary outcome measures
- Adequacy of the sample size calculation
- Adequacy of statistical methods used to compare groups for primary and secondary outcomes
- Relevant differences within target groups (gender, ethnicity, age and/or other relevant characteristics) and if applicable methods that will be used for additional (subgroup) analyses

Score: Excellent

Please indicate positive and negative aspects and address the major and minor negative aspects separately.

Positive aspects:

The application follows all international guidelines on TMS and has suitable details for the interventions, outcomes, sample size and statistical methods

Negative aspects:

There is no specific detail around how relevant differences within target groups (gender, ethnicity, age and/or other relevant characteristics) might be analysed, if relevant

2.4 Cost-effectiveness analysis

Please assess the cost-effectiveness analysis.

The purpose of the cost-effectiveness analysis (CEA) is to assess the proposed gain in health care efficiency of the (new) intervention(s) compared to the usual care provided in the Netherlands.

Consider the following factors:

- Whether all elements of PICOT (patients, intervention, comparator(s), outcomes, time horizon) are clearly stated in the cost-effectiveness analysis
- Whether the CEA is well designed
- Whether the methods of data collection are appropriate
- Whether the results are generalizable to other relevant settings
- For Dutch referees: is it consistent with the latest Dutch guidelines for health economic evaluations?

Score: Excellent

Please indicate positive and negative aspects and address the major and minor negative aspects separately.

Positive aspects:

This is not an area of expertise for me, but I found these sections well thought out and with good detail

Negative aspects:

The application recognises that not all clinics and patients have access to neuronavigation. Although it is stated that the Dutch health system will likely invest in neuronavigation in future, the cost may remain prohibitive to patients in low socioeconomic groups/geographical locations and it is not clear whether the SNT treatment can be made available equitably across the population

2.5 Budget impact analysis

Please assess the budget impact analysis.

The purpose of the budget impact analysis (BIA) is to assess the financial consequences of dissemination of the (new) intervention(s). Information obtained from the BIA can be used for policy decisions on a national, regional and/or local level. Various perspectives can be taken into account; the government and insurance perspectives must always be considered.

Consider the following factors:

- Clarity
- Well-designed in terms of perspectives, scenarios, population, time horizon, etc.?
- Are all relevant cost assessments included?
- Appropriate data collection?

- For Dutch referees: is it consistent with the latest Dutch guidelines for health economic evaluations?

Score:	Goed

Please indicate positive and negative aspects and address the major and minor negative aspects separately.

Positive aspects:

This is not an area of expertise for me, but I found these sections well written and they cover several different scenarios of roll out of the new protocol. The relatively long term follow up (25 weeks) seems to be a good idea as benefits will take time to appear.

Negative aspects:

Again, I think it would be useful to consider which groups might most benefit from the treatment and how the implementation might tackle social inequities in access to medical care.

2.6 Feasibility of the project

Please assess the feasibility of the study.

It is known that many projects in the Health Care Efficiency Research Program have a considerable delay and not always are the objectives achieved. In the grant proposal it should be motivated that the project is feasible within the proposed timeframe and budget.

Consider the following factors:

- Prospects of achieving the objective or objectives using this strategy
- Research protocol
- Realistic phasing and timetable
- Availability of facilities/staff
- Realistic number of patients/institutes/organisations
- Is the number of potential respondents to be approached in participating centres sufficient?
- Has the expected response been properly estimated? What is the non-response?
- Recruitment/participation of patients and if applicable: is randomisation feasible?

Score:	Excellent

Please indicate positive and negative aspects and address the major and minor negative aspects separately.

Positive aspects:

The research protocol is a very promising one and the team is skilled in all of the required disciplines.
The timing of the research is very realistic and takes into account the additional year required for data analysis and implementation. Overall the timeline of the project inspires confidence that the team are experienced and able to succeed in their implementation of the research outcomes.
The number of patients is good and takes into account attrition, and the multi-site aspect of the project is a real strength, giving insight into potential variability due to site-specific differences
The response rate if anything has been under-estimated as the applicants take a conservative approach based on response to ECT - if SNT performs as has been reported in published studies, then the response rate is likely to be higher and the benefits increase accordingly.
Randomisation is not feasible in this trial and the applicants have considered this in their experimental design and communication with participants.

Negative aspects:

None that I can see that have not been considered and addressed by the applicants

2.7 Systematic review

Please assess the systematic review.

See the appendix Systematic review.

Consider the following factors:

- Selection of search terms
- Are all relevant databases included?
- Selection of papers
- Are any references relevant to this specific proposal missing?

- Are the conclusions of the systematic review justified?

**Score:**	Excellent

Please indicate positive and negative aspects and address the major and minor negative aspects separately.

Positive aspects:

The literature on SNT is very small and applicants have identified the relevant publications

Negative aspects:

The literature is small, which raises some questions around the robustness of the outcomes, but the present application will make a significant impact on addressing the validity of the protocol by recruiting a much larger patient pool.

2.8 Project group

Please assess the project group.

Consider the following factors:

- **Relevant (multidisciplinary) expertise**
- **Familiarity with the discipline/research area**
- **Prior activities and products**
- **Ability of the group to successfully complete the proposed project**
- **Division of roles and tasks at implementation level**

**Score:**	Excellent

Please indicate positive and negative aspects and address the major and minor negative aspects separately.

Positive aspects:

Relevant (multidisciplinary) expertise: The team includes mainly psychiatrists but also an expert in Brain Stimulation, a statistician and patient representative (person with lived experience) and a health economist. The team has strong familiarity with the reserach area, as well as all of the expertise required for the project to succeed

The team has an outstanding track record of publications in fields relevant to the current project, and in particular I note that there are publications on cost effectiveness of interventions in the context of depression, providing evidence that the team has the required expertise and experience.

Negative aspects:

Division of roles and tasks at implementation level: the implementation plan is well described and follows evidence based guidelines. However the role of the team members in implementation are not clear

2.9 Overall quality assessment

Please assess the entire application with regard to the quality of the proposal based on the elements above.

**Score:**	Excellent

Please summarise your overall findings regarding the main strengths and weaknesses of the proposal. Please make sure your score is in line with your arguments.

Strengths:

This is a timely and relevant trial that will provide key clinical and cost benefit information about the validity of the SNT protocol of rTMS for depression. The study will be (to my knowledge) the first time that the SNT protocol has been adopted outside of the lab that first developed it, representing a significant contribution to validating this exciting treatment.

Weaknesses:

The lack of basic understanding of what SNT is doing to the brain remains a key weakness of the protocol and this application does not address the problem. However the protocol seems likely to deliver benefits to patients, and provide significant cost savings at many levels, making it a significant and beneficial study.

2.10 Budget

Please give your opinion on the budget based on the data supplied in the grant application. Important for the judgement of the budget is that usual care covered by Dutch health insurance is not chargeable to the grant. There is a comprehensive overview of the requested budget (in Dutch).

Please note that your assessment of these criteria should not be taken into account in your quality score above.

Consider the following factors:

- **The requested budget is realistic for this project proposal.**
- **The requested budget contains an extensive overview of activities that need to be funded.**
- **The requested budget (and co-financing) is clearly explained.**
- **The requested budget is appropriate given the nature of the product and the need for highly qualified staff.**
- **Cost-benefit ratio (the balance between effort, input, use of resources and the likely outcomes and yield)**

**Score:**	Realistisch

If possible, please indicate positive and negative aspects and address the major and minor negative aspects separately.

Positive aspects:

Budgets are never simple but the costs seem appropriate and justified. Inclusion of a PhD student is a positive as it provides an excellent training opportunity. Costings for treatment and expertise seem appropriate. Neuronavigation and MRI scans are required for the protocol so are necessary costs. Generous funding is allocated to the patient and end user engagement aspects.

Cost-benefit ratio is difficult to estimate because realistically that is one of the aims of the project. However, the approach is strong and well planned, and the expertise in the team will allow a robust cost-benefit ratio to be evaluated

Negative aspects:

Neuronavigation is funded only for 1 site so I assume that this means it is already available and in use at the other sites?

Beoordelingsformulier referent - DoelmatigheidsOnderzoek Open ronde 2023 UA 2022

Referentbeoordeling

1. Introduction

1.1 Details

**Grant programme:**	DoelmatigheidsOnderzoek
**Project number:**	10390012110079
**Applicant:**	Annemiek Dols
**Project title:**	Higher, faster, better: a study on (cost-) effectiveness of neuromodulation in depression. Is an accelerated intermittent Theta Burst Stimulation protocol compared to standard 10 Hz repetitive Transcranial Magnetic Stimulation, more (cost-) effective in patients with treatment-resistant depression?
**Code report:**	3704168

1.2 Conflict of interest and Confidentiality

Should you agree to assess this application, the Netherlands Organization for Health Research and Development (ZonMw) will assume that you can be regarded as an independent expert in the relevant field without personal interests as referred to in the Code for dealing with Personal Interests . If you have any doubts in this respect, please contact the programme team.

The procedures governing assessments require strict confidentiality. This means only that reviewers' assessments are always anonymized, but also that reviewers must handle all proposals strictly confidentially.

☑ I can be regarded as an independent expert, without any conflict of interest, in the relevant field, as referred to in the Code for dealing with personal interests https://www.zonmw.nl/en/about-zonmw/integrity-and-conflicts-of-interest/

☑ I declare that I will keep the content of the proposal and all additional information in this review process strictly confidential.

1.3 Instruction

For each section or sub-section, please describe both positive and negative aspects with regard to the listed criteria. For the overall quality assessment, please reread your comments in the boxes, give a score and summarise the main strengths and weaknesses of the proposal on which your score was based.

The following guidelines may help you when assigning scores while reviewing the grant application(s):

- **Excellent. The proposal successfully addresses all aspects of the criterion in question.**
- **Good. The proposal addresses the criterion in question well but certain improvements are necessary.**
- **Sufficient. The proposal generally addresses the criterion, but there are significant weaknesses that need corrections.**
- **Poor. The proposal shows weaknesses in relation to the criterion in question.**
- **Insufficient. The proposal shows serious weaknesses in relation to the criterion in question.**

Your review is an important contribution to the assessment of the application and will have a significant influence on the final funding decision. We would therefore greatly appreciate it if you could elaborate your scoring with in-depth comments and/or suggestions.

Please refer to the assessment criteria in such a way that your comments are consistent and informative for the applicant and the assessment committee.

The committee will make the final quality assessment based on the grant application, the reviewers' opinions and the applicant's response. The committee also assesses the relevance and priority of applications in terms of the programme specific criteria.

Your assessment will be sent to the applicant in anonymous form. They will then have an opportunity to respond. The committee will also receive an anonymous version of your assessment. We would, therefore, urge you to avoid making any references to yourself in your reviewer's report.

2. Reviewform

2.1 Objective and problem definition

Please assess the quality of the objective(s) and problem definition.

Consider the following factors:

- **How clear the objective is**
- **How clear and verifiable the problem definition is and whether it is consistent with the objective**
- **There is a clear description of the expected outcomes and intended results**
- **The value added to existing knowledge or practice**
- **The theoretical or empirical evidence presented in support of the problem definition**

**Score:**	Goed

Please indicate positive and negative aspects and address the major and minor negative aspects separately.

Positive aspects:

Clinical research project based on pilot data gathered by the applicant.
Clear scientific hypothesis and goals.
Should the study succeed, this would be a significant advancement for the field (science) and the patient population.
Scientific goals are clear and feasible.

This is a novel approach which, if successful, could be an adjunct in therapeutics , or at least open perspectives for further research.

Negative aspects:

My greatest concern regards scalability of the results of the present study. I believe the significance of the authors' findings will not be very clear, i.e. I am not sure one can envisage a situation where a clinician would refer for this treatment rather than (or in addition to) traditional clinical treatments.

2.2 Adequacy of the clinical study design

Please assess the adequacy of the clinical study design.

In the call for proposals extra attention is given to the adequacy of the chosen design in relation to the research question. Researchers should be able to explain how the proposed research method is appropriate for the question posed, demonstrate that the design will answer the research question, and why it is the most adequate approach. Researchers should specify the setting in which the research is conducted. For instance, is it feasible to randomize the patients if the research comparators are different in kind (e.g. surgery versus behavioral therapy)? Is it possible to have a control group using standard care?

Consider the following factors:

- **Is the chosen design well motivated in the grant proposal?**
- **Is the chosen design appropriate for the question posed and will this design answer the research question?**

**Score:**	Excellent

Please indicate positive and negative aspects and address the major and minor negative aspects separately.

Positive aspects:

The design is of excellent adequacy and well motivated

Negative aspects:

none

2.3 Methods of the clinical study

Please assess the methods of the clinical study.

Consider the following factors:

- **Eligibility criteria for participants**
- **Adequacy of the intervention for each group and description of the intervention with sufficient details**
- **Adequacy of outcomes defined as primary (patient oriented) and secondary outcome measures**
- **Adequacy of the sample size calculation**
- **Adequacy of statistical methods used to compare groups for primary and secondary outcomes**
- **Relevant differences within target groups (gender, ethnicity, age and/or other relevant characteristics) and if applicable methods that will be used for additional**

(subgroup) analyses

**Score:**	Goed

Please indicate positive and negative aspects and address the major and minor negative aspects separately.

Positive aspects:

The methodological aspects are very solid, and bases on pilot findings.

Negative aspects:

Relevant differences within target groups, more particularly ethnicity are less elaborated

2.4 Cost-effectiveness analysis

Please assess the cost-effectiveness analysis.

The purpose of the cost-effectiveness analysis (CEA) is to assess the proposed gain in health care efficiency of the (new) intervention(s) compared to the usual care provided in the Netherlands.

Consider the following factors:

- **Whether all elements of PICOT (patients, intervention, comparator(s), outcomes, time horizon) are clearly stated in the cost-effectiveness analysis**
- **Whether the CEA is well designed**
- **Whether the methods of data collection are appropriate**
- **Whether the results are generalizable to other relevant settings**
- **For Dutch referees: is it consistent with the latest Dutch guidelines for health economic evaluations?**

**Score:**	Excellent

Please indicate positive and negative aspects and address the major and minor negative aspects separately.

Positive aspects:

n/a

Negative aspects:

none

2.5 Budget impact analysis

Please assess the budget impact analysis.

The purpose of the budget impact analysis (BIA) is to assess the financial consequences of dissemination of the (new) intervention(s). Information obtained from the BIA can be used for policy decisions on a national, regional and/or local level. Various perspectives can be taken into account; the government and insurance perspectives must always be considered.

Consider the following factors:

- **Clarity**
- **Well-designed in terms of perspectives, scenarios, population, time horizon, etc.?**
- **Are all relevant cost assessments included?**
- **Appropriate data collection?**
- **For Dutch referees: is it consistent with the latest Dutch guidelines for health economic evaluations?**

**Score:**	Excellent

Please indicate positive and negative aspects and address the major and minor negative aspects separately.

Positive aspects:

see below

Negative aspects:

personnel cost is very high

2.6 Feasibility of the project

Please assess the feasibility of the study.

It is known that many projects in the Health Care Efficiency Research Program have a considerable delay and not always are the objectives achieved. In the grant proposal it should be motivated that the project is feasible within the proposed timeframe and budget.

Consider the following factors:

- **Prospects of achieving the objective or objectives using this strategy**
- **Research protocol**
- **Realistic phasing and timetable**
- **Availability of facilities/staff**
- **Realistic number of patients/institutes/organisations**
- **Is the number of potential respondents to be approached in participating centres sufficient?**
- **Has the expected response been properly estimated? What is the non-response?**
- **Recruitment/participation of patients and if applicable: is randomisation feasible?**

**Score:**	Excellent

Please indicate positive and negative aspects and address the major and minor negative aspects separately.

Positive aspects:

This study is founded in excellent pilot work and realistic in inclusion rates.

Negative aspects:

no comments

2.7 Systematic review

Please assess the systematic review.

See the appendix Systematic review.

Consider the following factors:

- **Selection of search terms**
- **Are all relevant databases included?**
- **Selection of papers**
- **Are any references relevant to this specific proposal missing?**
- **Are the conclusions of the systematic review justified?**

**Score:**	Excellent

Please indicate positive and negative aspects and address the major and minor negative aspects separately.

Positive aspects:

all relevant existing data are incorporated

Negative aspects:

none

2.8 Project group

Please assess the project group.

Consider the following factors:

- **Relevant (multidisciplinary) expertise**
- **Familiarity with the discipline/research area**
- **Prior activities and products**
- **Ability of the group to successfully complete the proposed project**
- **Division of roles and tasks at implementation level**

**Score:**	Excellent
**Please indicate positive and negative aspects and address the major and minor negative aspects separately.**	
**Positive aspects:**	
The project group has all expertise on board to complete the project successfully	
**Negative aspects:**	
none	

2.9 Overall quality assessment

Please assess the entire application with regard to the quality of the proposal based on the elements above.

**Score:**	Excellent
**Please summarise your overall findings regarding the main strengths and weaknesses of the proposal. Please make sure your score is in line with your arguments.**	
**Strengths:**	
project group is very specialised in this topic<br>good quality of pilot work<br>realistic and feasible	
**Weaknesses:**	
implementation strategy in group of clinical psychiatrists dealing with non-responsiveness	

2.10 Budget

Please give your opinion on the budget based on the data supplied in the grant application. Important for the judgement of the budget is that usual care covered by Dutch health insurance is not chargeable to the grant. There is a comprehensive overview of the requested budget (in Dutch).

Please note that your assessment of these criteria should not be taken into account in your quality score above.

Consider the following factors:

- **The requested budget is realistic for this project proposal.**
- **The requested budget contains an extensive overview of activities that need to be funded.**
- **The requested budget (and co-financing) is clearly explained.**
- **The requested budget is appropriate given the nature of the product and the need for highly qualified staff.**
- **Cost-benefit ratio (the balance between effort, input, use of resources and the likely outcomes and yield)**

**Score:**	Te hoog
**If possible, please indicate positive and negative aspects and address the major and minor negative aspects separately.**	
**Positive aspects:**	
n/A	
**Negative aspects:**	
Personnel costs are to me not clearly explained, certainly taken in to account the high budget foreseen	
